# Binding of HasA by its transmembrane receptor HasR follows a conformational funnel mechanism

**DOI:** 10.1007/s00249-019-01411-1

**Published:** 2019-12-04

**Authors:** Thomas E. Exner, Stefanie Becker, Simon Becker, Audrey Boniface-Guiraud, Philippe Delepelaire, Kay Diederichs, Wolfram Welte

**Affiliations:** 1 Pharmazeutisches Institut, University of Tübingen, Auf der Morgenstelle 8, 72076 Tübingen, Germany; 2 Department of Biology, University of Konstanz, Universitätsstr. 10, 78457 Constance, Germany; 3 Unité Des Membranes Bactériennes, Centre National de La Recherche Scientifique, Unité de Recherche, Associée 2172, Inst. Pasteur, 75005 Paris, France; 4 Institut de Biologie Physico-Chimique, UMR 7099, CNRS Université Paris Diderot 13, rue Pierre et Marie Curie, 75005 Paris, France

**Keywords:** Heme acquisition system, *Serratia marcescens*, Crystal structure, Outer membrane, HasR, HasA

## Abstract

HasR in the outer membrane of *Serratia marcescens* binds secreted, heme-loaded HasA and translocates the heme to the periplasm to satisfy the cell’s demand for iron. The previously published crystal structure of the wild-type complex showed HasA in a very specific binding arrangement with HasR, apt to relax the grasp on the heme and assure its directed transfer to the HasR-binding site. Here, we present a new crystal structure of the heme-loaded HasA arranged with a mutant of HasR, called double mutant (DM) in the following that seemed to mimic a precursor stage of the abovementioned final arrangement before heme transfer. To test this, we performed first molecular dynamics (MD) simulations starting at the crystal structure of the complex of HasA with the DM mutant and then targeted MD simulations of the entire binding process beginning with heme-loaded HasA in solution. When the simulation starts with the former complex, the two proteins in most simulations do not dissociate. When the mutations are reverted to the wild-type sequence, dissociation and development toward the wild-type complex occur in most simulations. This indicates that the mutations create or enhance a local energy minimum. In the targeted MD simulations, the first protein contacts depend upon the chosen starting position of HasA in solution. Subsequently, heme-loaded HasA slides on the external surface of HasR on paths that converge toward the specific arrangement apt for heme transfer. The targeted simulations end when HasR starts to relax the grasp on the heme, the subsequent events being in a time regime inaccessible to the available computing power. Interestingly, none of the ten independent simulation paths visits exactly the arrangement of HasA with HasR seen in the crystal structure of the mutant. Two factors which do not exclude each other could explain these observations: the double mutation creates a non-physiologic potential energy minimum between the two proteins and /or the target potential in the simulation pushes the system along paths deviating from the low-energy paths of the native binding processes. Our results support the former view, but do not exclude the latter possibility.

**Electronic supplementary material:**

The online version of this article (10.1007/s00249-019-01411-1) contains supplementary material, which is available to authorized users.

## Introduction

Most of our knowledge about protein structures is based on X-ray crystallography. While these crystal structures are accurate, they only provide lowest energy states occurring during the underlying molecular processes stable enough to be trapped in crystals. In contrast, molecular dynamics (MD) simulations starting and ending with crystal structures should in principle provide the full sequence of transient events. This way, experimental structures and simulations should be expected to complement each other and mechanistic studies should benefit from their combination. With this prospect, we worked on the heme acquisition system of the Gram-negative bacterium *Serratia marcescens* (Wandersman and Stojiljkovic 2000) comprising an outer membrane receptor HasR and an extracellular protein, the hemophore HasA. HasR belongs to the TonB-dependent receptors and consists of a 22-stranded membrane-spanning β-barrel closed by a plug. Heme-loaded HasA (holoHasA) binds to the receptor HasR and upon complex formation heme is irreversibly transferred from its HasA- to its HasR-binding site without energy input. HasR alone is still able to take up free heme down to a concentration of 500 nM, but together with HasA, which has a very high affinity both for heme and HasR (*K*_d_’s of 20 pM and 5 nM, respectively), the system is more efficient, allowing heme uptake down to a concentration of 10 nM (Rossi et al. 2003).

**Fig. 1:**
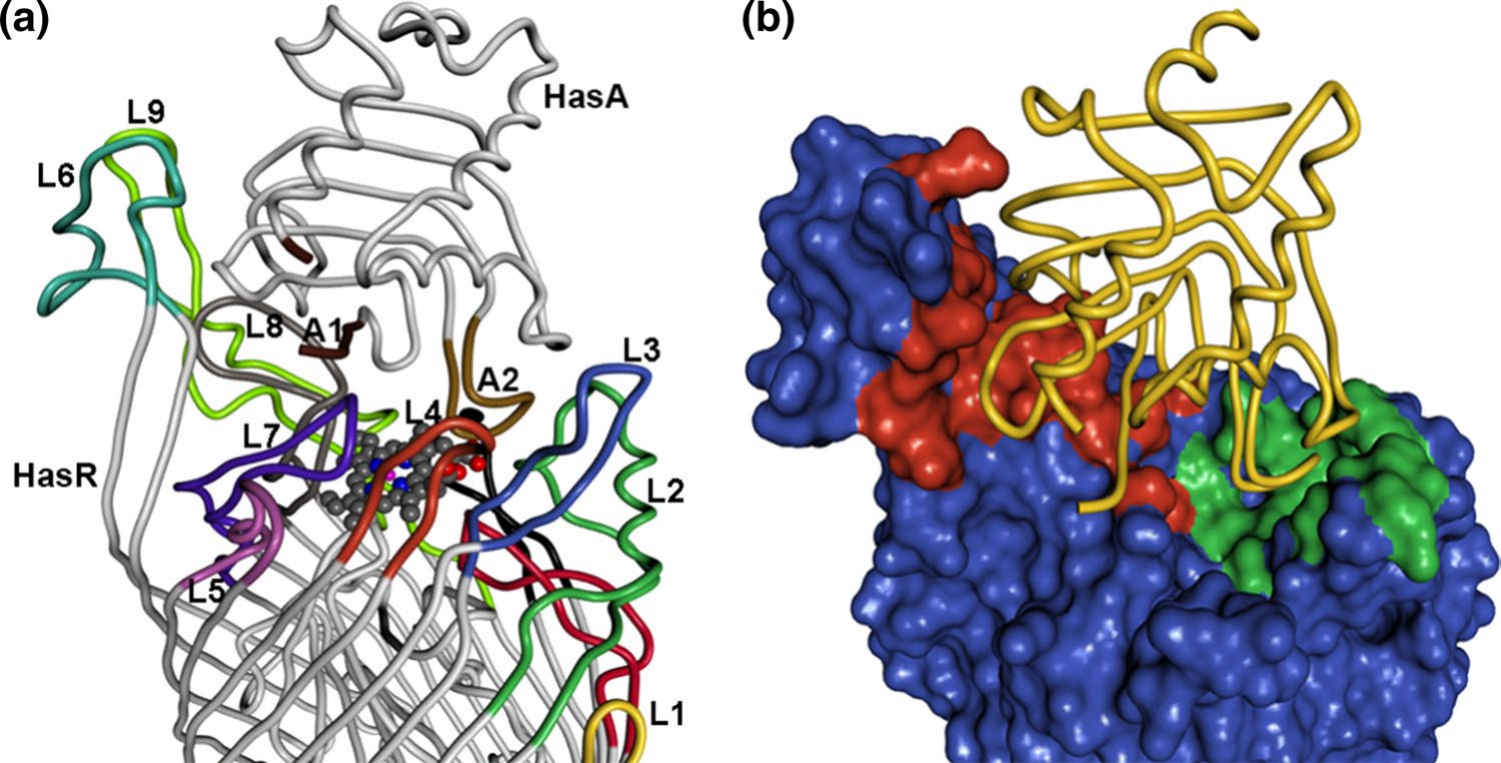
**a** Crystal structure of the complex of HasR with HasA (“WT complex”). Both proteins are shown in tube representation and heme is shown as ball and stick model. In the following, the orientations shown in this figure will be called “front view” and directions will be used according to these orientations. The loops (abbreviated as L) are color-coded as follows: HasR: L1: orange, L2: dark green, L3: light blue, L4: red, L5: magenta, L6: cyan, L7: dark blue, L8: gray, L9: light green, L10: black, L11: red, HasA, A1: dark brown (parts are unstructured in the crystal structure), A2: light brown. **b** Molecular surface of HasR with contact areas CA1 and CA2 in red and green, respectively. HasA is indicated in tube representation. Molecular graphic was made with the Pymol Molecular Graphics System, version 1.7.4

Both heme-free HasA (apoHasA) (Wolff et al. 2008) and holoHasA (Arnoux et al. 1999) structures are known and show a two-layered protein formed by alpha helices packed on a beta sheet, respectively. Two loops (A1 and A2) connect the two layers and define the heme-binding site, via two axial heme iron ligands, His32 on loop A1 and Tyr75 on loop A2 and a nearby His83 residue on loop A2. In the absence of heme, loop A1 is backfolded on the core of the protein. Upon heme binding, it undergoes a 30 Å displacement toward the edge of the core. The crystal structure of the wild type HasA~HasR~heme complex [PDB entry 3CSL (Krieg et al. 2009)], termed WT complex in the following, formed by incubating holoHasA with apoHasR, shows a very tight complex between the two proteins (see Fig. 1). HasA is oriented with its heme-binding site facing the receptor and heme is found translocated by 9 Å from HasA into its HasR-binding site, where two histidine residues form the axial ligands of the heme iron.

In the final complex, the interface between HasA and HasR covers 1745 Å^2^ and is mainly composed of two spatially separate areas: contact area 1 (CA1) consisting of loops L6, L8, and L9 from the receptor and contact area 2 (CA2) consisting of loops L2, L3, L10, and L11 (Fig. 1). The interface is highly polar (12 hydrogen bonds, 7 in CA1, 5 in CA2, 1 salt bridge in CA1), and the interaction between HasA and HasR is enthalpy-driven with a very high ∆*H* (− 40 kcal/mol). Previous experimental studies indicated that short deletions in either loop L6, L8 or L9 of CA1, which do not affect HasA-independent heme uptake, completely abolished HasA binding. In contrast, short deletions in loops L2, L3, L10, and L11 from CA2, did not impair HasA binding or free heme uptake, but HasA-mediated heme uptake (Barjon et al. 2007). This led us to hypothesize that the formation of the two contact areas, which are spatially separated, might also be time-shifted processes with CA1 formation starting before CA2 formation. However, the precise succession of events leading from the initial encounter to the late step of complex formation and to heme transfer is not deducible from the published crystal structures. To gain additional insight into this, we followed the approach outlined in the beginning, i.e. combining targeted molecular dynamics simulations and X-ray structure determination. We report the complex structure of HasA with a mutant DM-HasR, bearing two point mutations in the CA2 area. As expected, HasR forms a complex with HasA in the CA1 contact area. However, in contrast to our hypothesis, this DM complex of the two proteins was not seen in ten targeted MD simulations of the two proteins approaching each other from different non-bonded starting arrangements. In some simulations, CA2 contacts formed before CA1 contacts. None of them passed through the crystal structure. Only one simulation passed at a DM RMSD (see Abbreviations) of 3.45 Å. This suggested that the mutant HasR possesses a binding site for HasA, which is absent in the wild-type protein. Molecular dynamics simulations without target potential starting from the DM complex structure are in accord with this view. The limitations of the targeted MD approach on the other hand do not exclude the possibility that the DM complex structure is occurring as a transient in the native approach.

## Materials and methods

### Protein expression and structure determination

#### Molecular biology, mutant construction

The mutations were designed on the basis of the structure of the apoHasA~HasR~heme structure (WT complex structure 3CSL). A list of the polar bonds at the HasA-HasR interface is given in Table S1. The mutants were generated by PCR with mutagenic oligos (see Table S2) on pFR2 plasmid (Izadi et al. 1997). pFR2 harbours the genuine *hasR* sequence cloned into pBAD24 (Guzman et al. 1995) (including 42 base pairs after the TAA stop codon), taking advantage of the improved Shine-Dalgarno sequence present in pBAD24 and putting HasR expression under the control of the arabinose-inducible p_*araBAD*_ promoter present in the plasmid. In this construct, HasR is expressed without any tag.

CA1 mutants: five single mutants were made in CA1, namely S547A, S744A, Q668A, P669A, and S670A, as well as the double mutant S547A-S744A. S547 and S744 made the strongest H-bond to HasA residues (as estimated via the PISA server): Q668, P669, and S670 are in loop 8, very close to I671 that sits in the HasA heme pocket. Although P669 does not make a specific H-bond with HasA residues; we decided to mutate it due to its nature and its vicinity to the I671 residue.

CA2 mutants: four single mutants were made in CA2, namely R297A, N300A, Y308A, and N800A, as well as the double mutant hereinafter dubbed DM, R297A-N800A.

All mutations were verified by sequencing and recloned into an otherwise wild-type pFR2 plasmid with appropriate restriction sites. Double mutants were generated by exchanging the desired mutated restriction fragment from one mutant plasmid into the other one. The cloning strain was *Escherichia coli* XL1-Blue.

#### Biological tests

The mutant plasmids were transformed into *E. coli* C600 *∆hemA*, a heme auxotroph strain and growth of the mutants was assayed as previously described. Briefly, cells were grown in LB medium (supplemented with delta-aminolevulinic acid to bypass the effect of the mutation) at 37 °C up to an OD_600nm_ of 1, and then mixed with melted top agar and poured onto LB agar plates containing the appropriate antibiotics and arabinose at a concentration of 40 µg/ml to induce HasR receptor expression. Wells, punched with Pasteur pipettes were filled with heme sources (heme-albumin, holoHasA, or holoHasA plus heme-albumin at 10 µM concentration) at various dipyridyl concentrations (0, 50 and 150 µM) to induce the expression of the TonB complex. Plates were incubated overnight at 37 °C and scored for growth around the wells. All experiments were performed in triplicate. Figure S1 shows a representative experiment.

#### Protein purification

Mutants were transformed into *E. coli* popC4420 and growth was performed in 2 l or 4 l fermenters as previously described (Izadi-Pruneyre et al. 2006). All mutants were produced at wild-type levels and were purified in the same way as the wild-type protein. HasA was purified from *E. coli* pop3(pSYC150 + pSYC134/pAM) as previously described (Izadi et al. 1997). Heme loading of the protein was carried out with a ca 1.5 molar excess of hemin as compared to the protein. Hemin was provided as a concentrated solution (around 10 mM) in 0.1 N NaOH. A size exclusion chromatography step was included to eliminate excess heme.

#### Isothermal titration calorimetry (ITC)

Purified proteins (assumed to be 100% active in the HasA case) were extensively dialysed against 20 mM PBS, 150 mM NaCl, 0.02% Zwittergent ZW 3–14, and roughly adjusted to 100 µM and 10 µM, respectively, for the protein in the syringe (apo and holoHasA) and in the cell (HasR). ITC experiments were performed at 25 °C using the high-precision VP-ITC system (MICROCAL, GE HEALTHCARE, Worcestershire, UK) (Wiseman et al. 1989). Forty injections of 7 μl were injected into the 1.41 ml cell with a 10 min interval between injections. Heat signals were corrected for the heats of dilution and normalized to the amount of compound injected. The enthalpy of binding (Δ*H*), affinity constant (*K*_a_), and molar binding stoichiometry (*n*) were directly obtained from the titration curve fitted using the single site-binding model of the Origin7 software (ORIGINLAB).

#### Purification, crystallization, and structure analysis of the DM complex

The DM complex was formed and purified in the same way as the WT complex (Krieg et al. 2009) and crystallized under the same conditions (100 mM Tris pH 7–8, 1.8–2.2 M NaCl, 100 mM K_2_HPO_4_), but in a different space group (I222 with unit cell axes *a* = 102 Å, *b* = 115 Å, *c* = 261 Å) with only one DM complex per asymmetric unit.

HasR could be placed by molecular replacement using one chain from the WT complex complex as model with the program PHASER. However, PHASER failed to place HasA either from the complex or from holoHasA (1DK0), even though the density was clearly visible. One molecule from 1DK0 with heme was placed by hand in COOT, because strong positive density for the heme molecule was already visible in the density resulting from molecular replacement with HasR. The initial model was corrected by iterative rounds of refinement in PHENIX and manual correction in COOT. The data statistics and refinement statistics are given in Table 1.

**Table 1:** Data collection and refinement of the DM complex structure

Data collection		Refinement	
Space group	I222	Resolution range (Å)	37.75–2.79 (2.87–2.79)
Unit cell parameters (Å)	*a* = 101.9	Number of reflections	37,807 (2458)
	*b* = 114.6	Completeness (%)	98.8 (89.0)
	*c* = 260.7	Rwork	0.2417 (0.453)
		Rfree	0.2964 (0.474)
Wavelength (Å)	1.00	No. of amino acid residues	840
Resolution range (Å)	40–2.79 (2.96–2.79)	No. of heme atoms	43
I/σ	13.26 (0.91)	Deviation from ideal bond length (Å)	0.003
Reflections	250,154 (37,099)	Deviation from ideal bond angles (°)	0.667
Completeness (%)	99.0 (94.9)	Ramachandran favoured (%)	88.6
Redundancy	6.6 (6.4)	Ramachandran allowed (%)	98.8
Rmeas (%)	10.3 (242.1)		
CC1/2	99.9 (70.7)		

Values in parentheses correspond to the highest resolution shell

### Molecular dynamics simulations

#### Model generation

The crystal structures of the apoHasA~WT–HasR~heme complex (PDB code 3CSL, termed ‘WT complex’ or ‘WT complex structure’ in the following) and of the holoHasA~DM–HasR complex as described below (PDB code 5C58, termed ‘DM complex’ or ‘DM complex structure’ in the following) were the basis for all simulations. 3CSL lacks the HasA-loop A1 that could not be modeled during structure determination due to weak electron density. To generate a complete reference structure of HasR alone, the loop was modeled using the program Prime from the Schrodinger Package 8 (2015; Jacobson et al. 2004). This and the other missing loops in the DM structure were added using the Modeller 9.10 software using 3CLS as template for the loops and 5C58 for the rest of the proteins and the heme molecule. Protons were added at the riding positions using the Xleap module of the AMBER 10 program package (Case et al. 2008), except for the histidine residues coming in contact with the heme. Here, protonation of the ε-nitrogen was precluded. The Cornell et al. force field (parm96) (Cornell et al. 1995) was used for calculating potential energies of the proteins and ions. The parameters for heme were taken from D.A. Giammona (Cornell et al. 1995; Giammona 1984). The system was placed in a rectangular box of TIP3P water molecules. The borders were chosen to be at least 10 Å from every solute atom. Twenty two Na^+^ ions were added for charge compensation. No additional ion concentration was used to keep salting out effects to a minimum. To minimize the computational demand, no explicit membrane model was used. Instead, restraints onto the backbone of the β-barrel were imposed (see below) to increase the stability and avoid unrealistic conformational changes in this part of the model.

#### Heating and equilibration

The simulations presented here were conducted using the Amber10 molecular dynamics simulation package (Case et al. 2008). Numerical integration of the equations of motion used a time step of 2 fs with atomic coordinates saved every 1.0 ps. SHAKE (Ryckaert et al. 1977) was used for geometry restraints on all protons. All systems were minimized for 10,000 cycles; the first 50 using the steepest descend method and conjugate gradient for the rest. The system was then heated up from 100 to 300 K during 1 ns using the Berendsen thermostat (Berendsen et al. 1984) with a coupling constant of 1.0 ps (NVT ensemble) followed by 1 ns pressure regulation to 1 bar using isotropic position scaling with a coupling constant of 1.0 ps (NPT ensemble).

During these stages, geometrical restraints of 5.0 kcal/(molÅ^2^) were imposed on the backbones of both proteins. These restraints were then removed for all atoms except the C_α_ atoms of the transmembrane β-barrel in a series of five short MD simulations of 0.05 ns each. The restraints on the β-barrel were used to approximate the stabilizing effect of the membrane on this part of the system. The simplifications are justified for the following reasons:

(1) We simulated here the approach and binding of HasA to the extracellular part of HasR and assumed that the influence of the transmembrane part on this process can be neglected. (2) Membrane parameters are available only for standard lipid double layers; HasR however, resides in the bacterial outer membrane with a lipopolysaccharide (LPS) membrane leaflet facing the extracellular side and protruding with their oligosaccharides far into the medium. (3) The β-barrel itself is very rigid and showed no significant changes during unrestrained simulations (data not shown).

The system was then allowed to equilibrate for another 1 ns. In the preparation of the targeted MD runs (see below), a very small restraint of 1.0 kcal/(molÅ^2^) was imposed on C_α_ atoms of HasA to keep it close to the starting structure and prevent all interactions with HasR. The final structures were then used as starting points for the production runs.

#### Free simulations

Production runs were performed for the free HasR and the HasA~HasR complex starting from the DM structure without any guiding potential and constraint only on the transmembrane β-barrel of HasR to avoid artifacts of the missing membrane. These used constant volume at 300 K and periodic boundary conditions (NVT ensemble). To better sample the conformational space, five independent simulations were conducted using the same starting arrangement, but different randomly chosen velocities at the start of the heating period (see above).

#### Targeted MD

Ten starting arrangements of holoHasA and HasR for modeling complex formation were constructed by manually displacing HasA approximately 20 Å away from its final position in the complex. All production simulations of the approach of HasA toward HasR were performed using constant volume at 300 K and periodic boundary conditions (NVT ensemble). The targeted MD (Schlitter et al. 1994) was used to force HasA toward its end structure in the binding site of HasR by reducing the target RMSD between the current simulated systems and the end structure using an artificial target potential. This target potential is chosen to be proportional to the square of the difference between the current RMSD calculated from the atomic coordinates at a specific time point and the target RMSD, a value chosen by the user for each time point to control the speed of the forced approach of the final structure. For the ten starting arrangements, the RMSD to the WT complex was calculated. During the period of the planned simulation, this starting target RMSD value was reduced linearly to zero (Aci-Seche et al. 2011) and these values were used as target RMSD values at the respective current time. A current RMSD value is calculated from the instantaneous arrangement of HasA and different substructures of HasR, onto which the guiding potential acts (called target masks in the following), the optimal selection of the target mask was determined in short test runs (data not shown). The backbone non-hydrogen atoms, as well as Cβ and Cγ (if available) of the loops building the contact areas of HasA, as well as HasR were found to yield simulations in which all important interactions were formed without overconstraining the simulation and were selected for the production runs. With this selection, starting target RMSD values with the atoms in the target mask, i.e. the atoms that are forced toward their location in the WT complex, were determined and yielded values between 16 and 24 Å for the ten simulations. These were then reduced by 1 Å every 0.4 ns and 1.0 ns in fast and slow simulations, respectively.

**Fig. 2:**
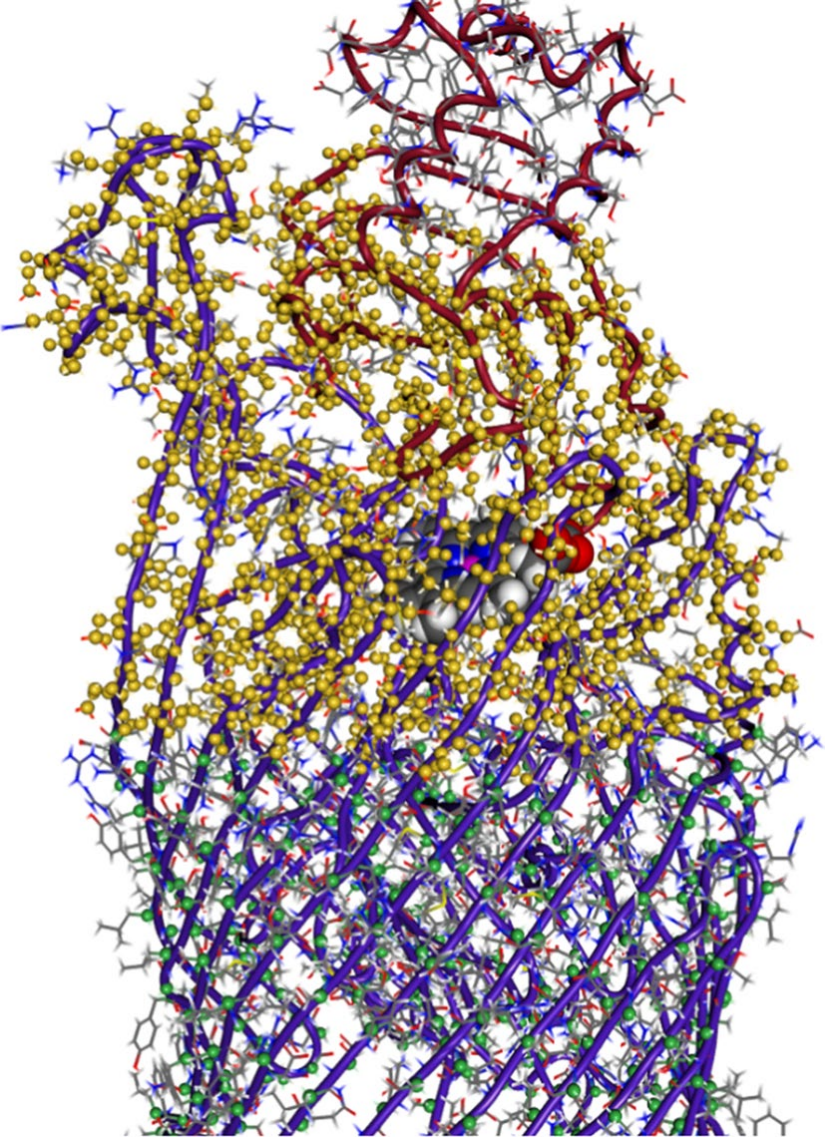
Target mask. The WT complex served to select the target mask atoms and constraints for the targeted MD simulations. The C_α_ atoms of the barrel, on which position constraints were imposed to model the influence of the membrane, are marked in green. Atoms of HasR and HasA which formed the target mask to which the simulated structure is slowly forced in the targeted MD simulation are marked in yellow

By the linear descent of the target RMSD, the current arrangement of the proteins is pushed toward the crystal structure. In normal targeted MD simulations, the current RMSD values are calculated after rotating and translating the system into the reference coordinate system of the target structure. This is needed because, the molecules are moving and tumbling in the simulation due to thermal fluctuations and the current RMSD would be dominated by these transitions. Since position constraints were applied onto the β-barrel of HasR to approximate its membrane integration, the coordinate system of the simulation will always coincide with this reference frame and no re-alignment is needed to describe the binding process accurately. The atoms of this target mask are shown in Fig. 2.

The different RMSD values are listed in the Abbreviations.

Simulation runs during a 6 ns period, termed ‘short simulations’ in the following, were started with the ten different starting arrangements of HasA relative to HasR. After equilibration following the standard protocol described above, HasA is guided into its location in the crystal structure by steadily reducing the target RMSD by 2.5 Å per ns. A force constant of 1.0 kcal/(molÅ^2^) for the guiding potential was applied. To reduce the speed of the approach and in this way limit the target force and simulating a more realistic system, long simulations were additionally performed starting from an intermediate of the short simulations reducing the target RMSD by 1 Å per ns.

#### Calculation of target energy

Since the restraint energy given in the output of the MD simulation is the sum of the target energy and the position restraint energy of the transmembrane region of HasR, the target energy is not directly obtainable. Thus, additional single point calculations were performed on each snapshot stored in the trajectory file including the guiding potential, but not the position restraints.

#### Molecular graphics

Molecular graphics were made with Molcad II, V1.4, MOLCAD GmbH, Darmstadt, Germany (Brickmann et al. 2000), except for Figs. 4, 5, which were made with Pymol.

## Results

### Screening of binding requirements by mutants and characterization by isothermal calorimetry

Analyses of the wild-type complex structure (WT complex in the following) showed that HasR loops from both contact areas contribute to the binding of HasA (Fig. 1). Mutational studies revealed that all loops, except the very short ones L1 and L4, are needed to support HasA-mediated heme uptake (Barjon et al. 2007). Thus, we hypothesized that both contact areas are necessary to hold HasA in the correct position for heme transfer, which is in addition dependent upon the Ile671 side chain of HasR in the heme-binding pocket of HasA. To test this, we used the crystal structure to spot HasR residues in each contact area which are noticeably interacting with HasA (see Table S1). Accordingly, site-directed single mutants and a double mutant of HasR were constructed and tested by growth assays for their heme acquisition properties in an *E. coli* heme auxotroph strain, with heme alone, with holoHasA, or with holoHasA and heme (all at 10 µM), and at three dipyridyl concentrations to allow for a variation of TonB complex concentration (Figure S1). It was previously shown that a high TonB complex concentration is required for HasR to be able to acquire heme from HasA. At low TonB complex concentrations, HasA inhibits free heme acquisition by HasR (Ghigo et al. 1997).

Five single mutants were constructed in CA1 [Ser547Ala (L6), Gln668Ala (L8), Pro669Ala (L8), Ser670Ala (L8), and Ser744Ala (L9) and four in CA2 (Arg297Ala (L3), Asn300Ala (L3), Tyr308Ala (L3), and Asn800Ala (L10)]. None of the single mutants designed to abolish H-bond between a receptor residue and a HasA residue appeared to have a distinct heme acquisition phenotype in our in vivo growth tests. Hence, we constructed a double (Arg297Ala, Asn800Ala) mutant, referred to as DM-HasR in the following. These two residues in CA2 are making hydrogen bonds with the backbone of the HasA heme-binding loop A2, suggesting that loop A2 would no longer be held in a stable position vis à vis the receptor. In the growth tests, DM-HasR was able to take up free heme as efficiently as WT, but it could not take up heme from holoHasA (see Figure S1, plate C, well 2). It formed a stoichiometric complex with holoHasA (Table 2), which showed a holoHasA-like absorption spectrum (Figure S2), strongly indicating the absence of heme transfer from HasA to HasR, as previously found in Ile671Gly.

**Table 2:** Thermodynamic parameters of the interactions of apoHasA and holoHasA with the WT and mutant HasR receptors, as deduced from the ITC experiments

	HasA (apo)				HasA (holo)			
	*N*	*K*_a_ (M^−1^)	∆*H* (kcal × M^−1^)	∆*S*(cal × M^−1^ × K^−1^)	*N*	*K*_a_	∆*H*	∆*S*
DM-HasR	1.05	1.53 × 10^7^	− 26	− 55	1.02	2.4 × 10^6^	− 15	− 22
Ile671Gly	1.06	1.8 × 10^7^	− 54	− 116	0.99	9.4 × 10^7^	− 46	− 120
WT-HasR	0.92	1.7 × 10^8^	− 43	− 106	1.11	1.1 × 10^6^	− 21	− 42

*N*, *K*_a_, Δ*H* and ∆*S* correspond to the stoichiometry of the reaction, the association constant, the enthalpy variation and the entropy variation, respectively

**Fig. 3:**
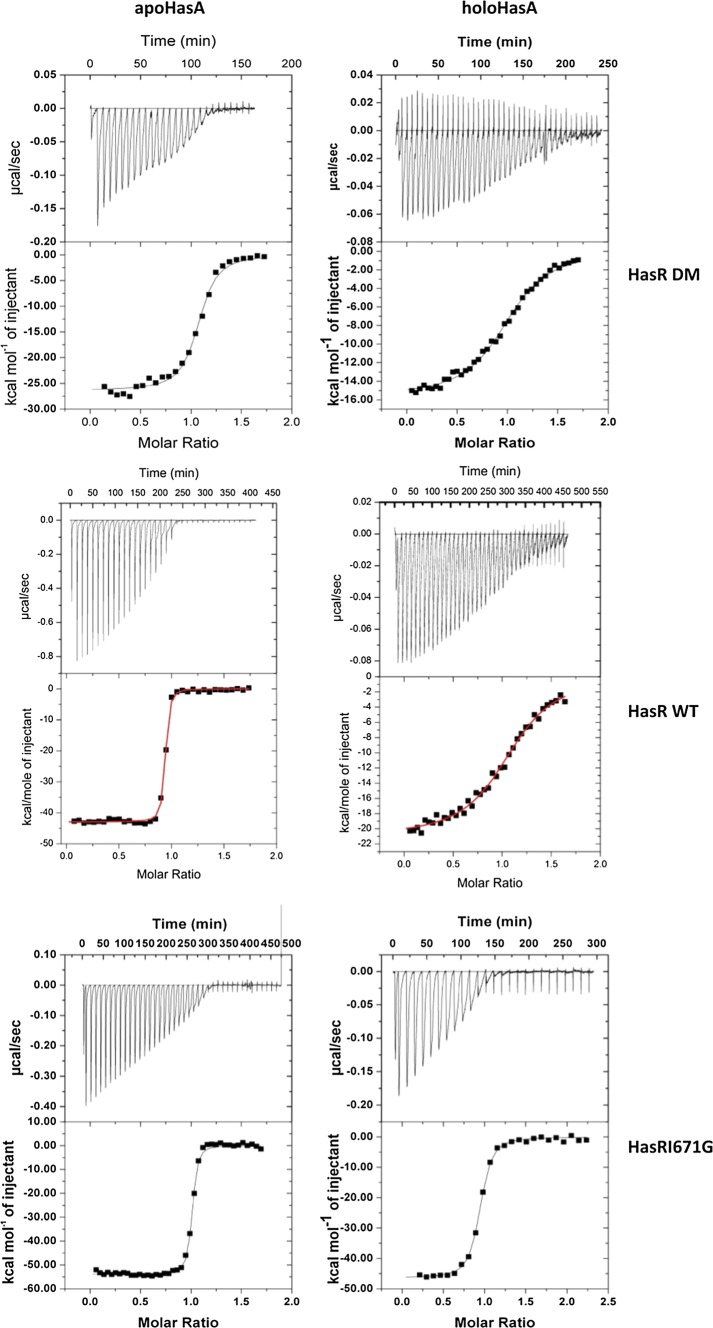
ITC titration of DM-HasR, WT-HasR, and Ile671Gly-HasR with apoHasA and holoHasA. ∆*H* was determined from the integration of the different peaks and *K*_a_ from the slope at the inflexion point

ITC data (Fig. 3 and Table 2) hint to striking differences in the interaction of holoHasA with DM-HasR and Ile671Gly-HasR (− 15 kcal/mol) and (− 46 kcal/mol), indicating a strong deterioration of the interactions in the former. Further, *K*_d_, as calculated from *K*_a_, for holoHasA dissociation from DM-HasR is higher by a factor of around 50.

According to these results, holoHasA forms a one-to-one complex with DM-HasR, but the two residues modified in CA2 are critical for achieving a high binding strength. According to the growth data and because the dissociation of heme from HasA will cost energy, this may be necessary in the preparatory stages of heme transfer. Yet, tight complex formation alone is not sufficient for heme transfer as the Ile671Gly-HasR mutant shows. In the latter complex, heme transfer might be blocked at a later stage of heme transfer.

To understand these profound differences in the phenotype of DM-HasR as compared to WT-HasR and Ile671Gly-HasR, we determined its crystal structure in complex with holoHasA.

### The DM complex structure

The final model (PDB code 5C58) contains one HasA, one HasR, and one heme molecule. The conformation of the HasR barrel and of the plug is the same as in the WT complex. However, the extracellular loops L2, L3, L10, L11 forming CA2 and loop L4, as well as parts of L9 from CA1 could not be modeled due to missing electron density. L4 by itself plays no role in either HasA or heme binding and displays the highest B-factors in the WT complex 3CSL structure. Those long loops are likely to be quite flexible as long as they are not trapped in one conformation upon HasA binding. Additionally, the electron density of the two loops of HasR bearing the heme-coordinating histidines (L7 of the barrel bearing His603, and apex C of the plug bearing His189) are much less well defined than in the WT complex. Finally, L6 which is part of CA1 is bent away toward the “exterior”.

**Fig. 4:**
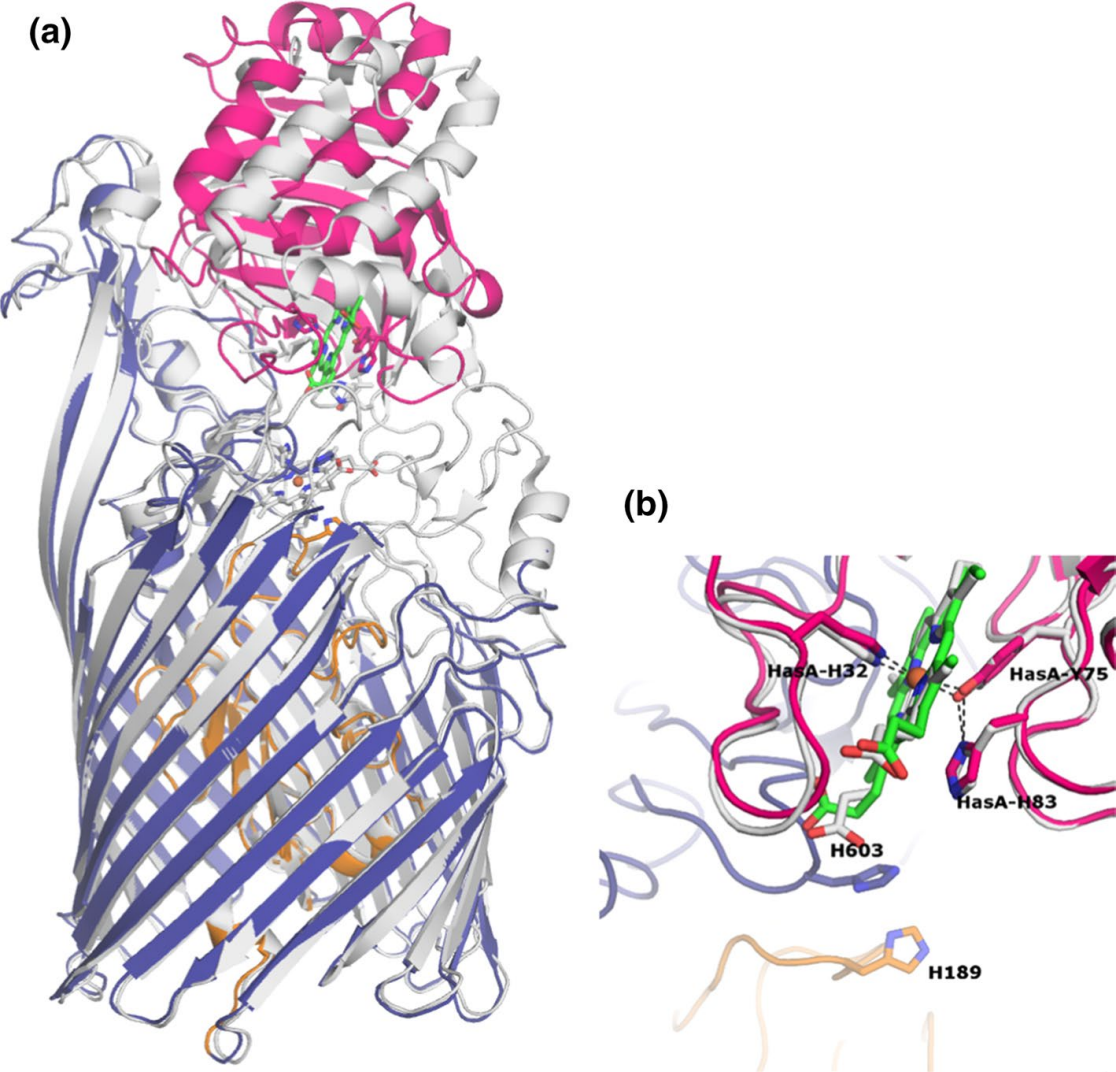
Overlay of the DM complex (HasA in pink, HasR barrel in blue, HasR plug in orange) and the WT complex (HasA and HasR in grey). **a** Overview in ribbon representation; **b** Heme binding site compared to that of the holoHasA structure (PDB code 1DK0, gray)

HasA in the DM complex is in the same conformation as in the holoHasA structure (Arnoux et al. 1999), 1DK0, i.e. the heme is coordinated by residues His32 (loop A1) and Tyr75 (loop A2) from HasA (see Fig. 4b). A superposition of the DM complex with the WT complex shows that HasA is further away from HasR and tilted in the former with respect to its position in the WT complex (see Fig. 4a). The interface area is smaller (1043 Å^2^ in the DM complex compared to 1745 Å^2^ in WT) and is restricted to CA1, with two hydrogen bonds made by L6, three by L7, four by L8 (only 4 with a distance shorter than 3.5 Å), and one salt bridge by L9. These bonds differ from those found in the WT complex, with the exception of the HasA Asn62–HasR Ser547 bond. A complete list of hydrogen bonds and salt bridges for both structures can be found in Table S1.

### Relevance of electrostatic interactions for the binding of HasA to HasR

**Fig. 5:**
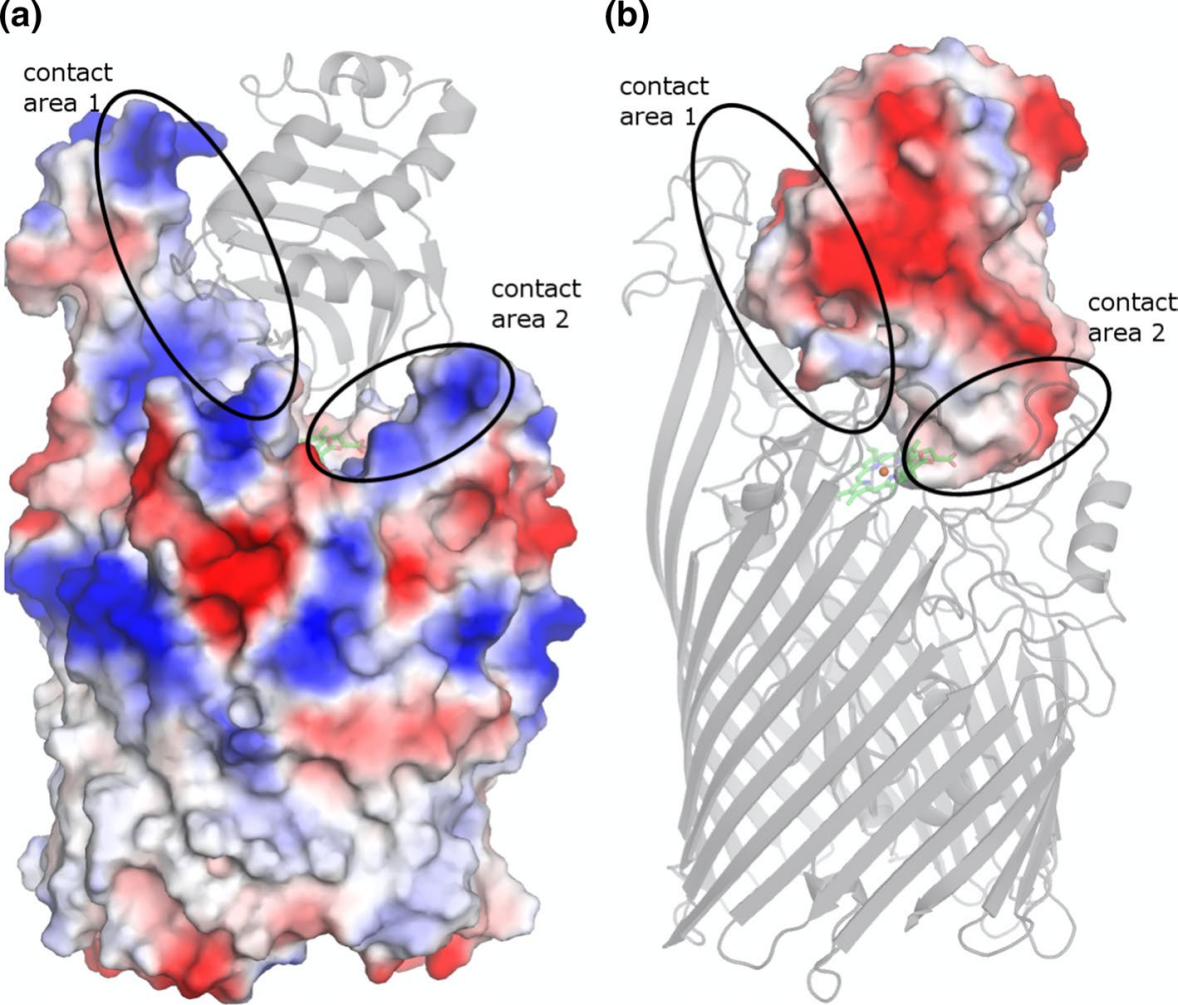
Electrostatic surfaces of the WT complex as calculated with the APBS plugin (Baker et al. 2001) of the Pymol Molecular Graphics System, version 1.7.4 (positive potential blue, negative red). **a** HasR with a pale ribbon model of HasA; **b** HasA with a pale ribbon model of HasR

The two contact areas of HasR for binding of HasA have a distinctly positive potential (see Fig. 5a) due to a total of 16 basic residues (9 Lys and 7 Arg) and only five acidic ones. Four lysines/arginines are located at the top of the long protruding loops L6–L9 at the external border of CA1. One arginine residue is located at the top of L3. The electrostatic potential reaches into the extracellular medium. In contrast, the surface of HasA contains 15 acidic residues, constituting almost 10% of the whole protein conferring a negative surface potential to it (Fig. 5b).

These complementary potentials likely generate a driving force to steer the two proteins together, when they are both at non-contacting distances (Schreiber and Fersht 1996).

The structure of the DM complex is in accord with the much weaker interaction of holoHasA as compared with Ile671Gly-HasR reflected in the respective *K*_a_ values obtained from ITC data (see Table 2). Three interpretations of the DM complex are possible:

It is a necessary transient arrangement of holoHasA with HasR during the progression of arrangements leading to the WT complex formation.

It might occur in only one out of several alternative reaction paths leading to the WT complex.

It could be absent from all of these alternative reaction paths, because of the non-native interactions between holoHasA and HasR caused by the mutations.

Because experimental techniques to track the native reaction paths are unavailable, we used molecular dynamics simulations to discriminate between these interpretations.

### Characterization of the dynamic behavior of the DM complex structure and the same structure without the mutations

The crystal structure showing holoHasA bound to DM-HasR appeared as a candidate of a necessary intermediate state in the formation of the final WT complex. Because this hypothesis involves the re-interpretation of a static crystal structure of a mutant as a dynamic intermediate, we undertook simulations of the complex as it is and with the same structure but without the two mutations.

After the heating, pressure relaxation, and equilibration steps, changes in the structures and the relative orientation of HasA and HasR were followed for 100 ns during the production run. To avoid a bias by forcing the flexible, invisible loops of DM-HasR in the DM complex into their corresponding WT complex orientation, the loops were relaxed during a 50 ns simulation in which the C_α_ positions of HasR and HasA other than the flexible loops were constrained to their coordinates in the DM structure. HasA was then allowed to move freely in the 100 ns production run. For better statistical sampling, five independent simulations were performed for both systems.

**Fig. 6:**
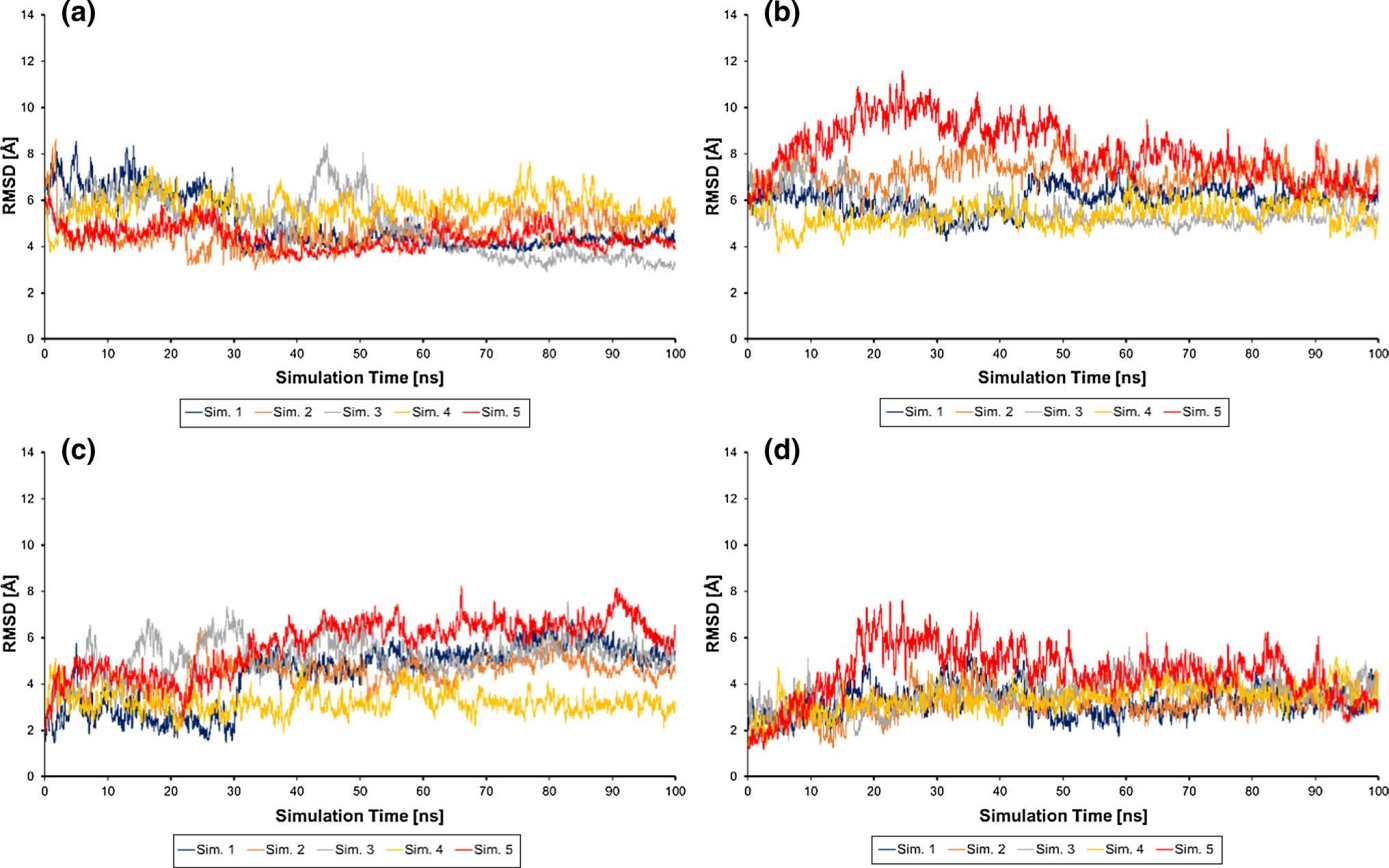
**a**,** c** The holoHasA~WT-HasR complex in free simulations starting from the DM structure. **b**, **d** The holoHasA~DM-HasR complex in free simulations starting from the DM structure. **a**, **b** C_α_ RMSD of holoHasA to the WT complex; **c**, **d** C_α_ RMSD of holoHasA to the DM complex

In the holoHasA–WT-HasR simulations, holoHasA is moving in four from five simulations away from the DM complex (Fig. 6c) and approaches the WT complex structure with an RMSD between 3 and 4 Å (Fig. 6a) to the final structure interfacing contact site CA1 and CA2. Especially, the gray simulation shows at the end very good similarity with the WT structure, but also the blue and red simulations come close to it. One exception is the yellow simulation, where the DM complex structure is stable over the complete simulation. The orange simulation is, after first approaching the correct binding conformation, leaving this structure again and adopts a final structure, which is different to the DM complex and WT complex structures.

It can be concluded that the DM complex structure for the holoHasA–WT-HasR system is metastable. It represents a local minimum, in which the yellow structure stays for the complete simulations and the others also have to overcome a barrier first. When these latter simulations have passed the barrier, the undisturbed contact area CA2 in the wild-type funnels holoHasA to its final binding site even if the experimental WT complex structure is not reached.

In contrast, the holoHasA–DM-HasR system is moving in none of the simulations toward the WT complex structure (Fig. 6b). In four simulations, the DM complex stays more or less stable. The final RMSD is still under 4 Å, which is acceptable for such large systems and can be considered as structures in the same minimum. Only in the red simulation, the structure is visiting other arrangements, but these correspond to neither the DM complex nor the WT complex structures. At the end, even this simulation is going back to a structure close to the starting DM complex structure (Fig. 6d).

As summary of these unrestrained simulations, the RMSD values found in the holoHasA–WT-HasR system suggest that it experiences only a small potential barrier against leaving the DM complex structure and develops in four of five simulations toward the WT complex structure. This is probably due to the undisturbed CA2 loops, which establish the correct orientation and electrostatic interaction for a smooth transition. In contrast, the holoHasA–DM-HasR system does not develop toward the WT complex and remains trapped close to the DM complex structure. The distorted CA2 cannot establish the driving force to drag holoHasA from its binding position in the DM complex to the final location needed for heme transfer. Hence, the stability of the DM complex, both under physiologic conditions and in the dynamic simulation, is caused by the DM mutations.

### Simulating the binding events

The simulations showed that the holoHasA–WT-HasR system is not forming a stable DM complex structure in accord with the physiologic system. We now addressed the questions whether the DM complex structure is a necessary transitional state of the binding process or if there are alternative reaction paths, whether the DM complex is visited in some of these. To answer this, simulations of the full binding process were performed. Even with the extremely high binding affinity of holoHasA to WT-HasR, such simulations still impose significant demands on the sampling method, both with respect to the length of the simulations and the number of parallel simulations starting from different relative orientations of the two unbound proteins. Simulations up to the millisecond to second time scale have been reported (see e.g. Dror et al. 2010 for an early example). However, these consume a significant amount of computer power even with today’s acceleration techniques and require dedicated hardware. Moreover, such long simulations do not guarantee the appearance of desired rare events like complex formation (Markwick and McCammon 2011; Schlick 2009; Spiriti et al. 2012; Gedeon et al. 2015; Elber 2005). To overcome these limitations and at least approximately characterize rare events, artificial biasing potentials have been introduced to force the simulation in the functional direction and to hinder it from venturing into irrelevant regions of the phase space, e.g. the ligand diffusing away from the binding site. Here, we used targeted MD (Schlitter et al. 1994) to force the system toward a given end structure by reducing the target RMSD between the simulated systems or parts of it and the end structure. In the simulation described here, the backbone heavy atoms, as well as C_β_ and C_γ_ (if available) of the loops building the contact areas of HasA, as well as HasR were chosen as the subsystem called target mask in the following, on which the additional target forces were applied (see Fig. 2). For each step of the simulation, a target RMSD value is specified, which is compared to the current RMSD between the simulation and the end structure of all atoms in the target mask. The artificial target potential is then set to be proportional to the square of the difference between the target RMSD and the current RMSD. During the simulation, the target RMSD is lowered linearly to zero and the current RMSD follows it pushed by the forces resulting from the current target potential. The size of the current target potential represents potential barriers which the system experiences.

**Fig. 7:**
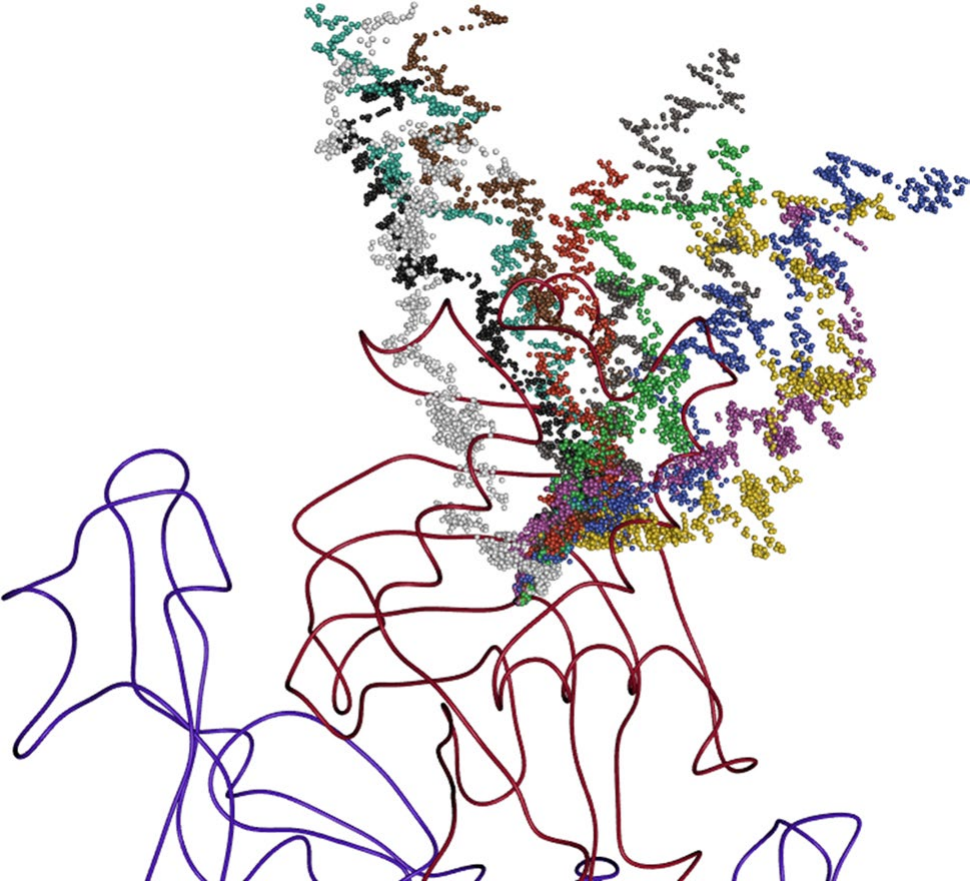
Paths seen in the ten targeted MD simulations visualized by representing the center of mass of holoHasA as small spheres: Sim1: red, Sim2: green, Sim3: blue, Sim4: yellow, Sim5: magenta, Sim6: cyan, Sim7: brown, Sim8: grey, Sim9: white, Sim10: black. For reference, the end structure of holoHasA and HasR is shown in tube representation in red and blue, respectively

Ten different starting arrangements were generated by displacing HasA from the binding site of HasR in the WT complex. These are shown in Fig. 7 and Figure S6 and were chosen to cover approximately uniformly the space above HasR in the “front view” as introduced in Fig. 1. These starting arrangements have initial current RMSD values to the final WT complex of all atoms in the target mask between 16 and 24 Å. After equilibration, HasA was guided into its location in the WT complex by steadily reducing the target RMSD first by 2.5 Å/ns (short simulations). When the target RMSD reached a value of 10 Å, the reducing rate was lowered to 1.0 Å/ns, referred to as short simulations in the following. Before dropping below this threshold, HasA diffused through the solvent and only started to form the first weak pairwise contacts with HasR. From then on, interactions formed in the two contact areas. Because complex formation of the final WT complex and very likely also of intermediate arrangements before relies on specific interactions, the conformational space needs to be searched more rigorously. The lowered target RMSD reducing rate grants more time to the system to find these interactions and avoid venturing into unrealistic, high-energy arrangements. The paths of HasA followed in the simulations are visualized in Fig. 7 by points representing the center of mass of the protein (individual paths are also presented in Figure S6 of the Supporting Material with two different orientations). It can be clearly seen that different starting arrangements lead to large variations in the early stages of HasA approaching HasR. The upper part of the figure corresponds to free diffusion of HasA, which is only slightly biased by the target potential to move into regions closer to HasR. At approximately half of the initial RMSD, HasA adopts more stable poses, which can be seen by groups of points close in space. This is caused by local potential energy minima, i.e. transient interactions with HasR, which have to be broken again for further approach of HasA toward the end structure. Which intermediates are visited by a simulation depends strongly on the starting arrangement. Only in the final stages of the complex formation do the points in Fig. 7 restrict to narrower regions in space and finally they all converge toward the WT complex.

**Fig. 8:**
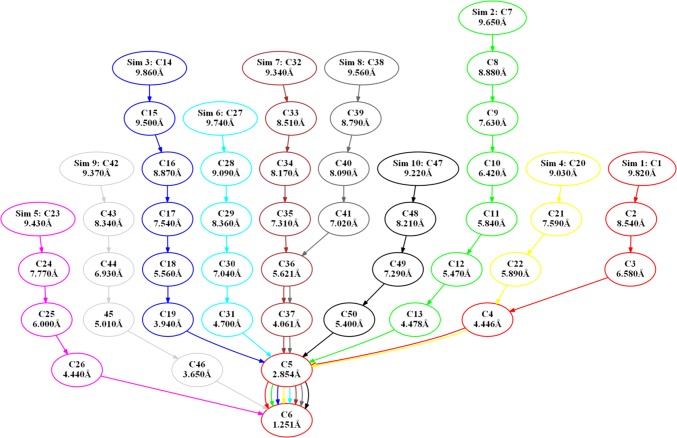
Graph presentation of the clustering. Consecutive clusters of one simulation are connected by arrows and coded by color. Each cluster can be interpreted as an intermediate and the path from the top to the bottom as a possible path of complex formation. Simulation 5 and 9 do not share clusters with other simulations except for the final cluster. Simulations 7 and 8 are most similar sharing 4 clusters followed by simulations 1 and 4, which share 3 clusters. The Å value of each cluster indicates the average target RMSD of the cluster arrangements. The color code is the same as in Fig. 7

The arrangements of the simulations seem to cluster and converge. The clusters are putative intermediate metastable arrangements. To quantify these visual impressions, snapshots of all simulations were combined and clustered using the PTRAJ tool from the AMBER software (Roe and Cheatham 2013). To exclude the free diffusion, we limited the analysis to the parts of the simulation, where the target RMSD had dropped below 10 Å, i.e. to the phase of slow target RMSD reducing rate. Setting the number of generated clusters to 50 gave a good representation of intermediate arrangements seen in Fig. 7. The clusters obtained are schematically shown in Fig. 8. The average RMSDs between the arrangements in one cluster ranges from 0.6 to 1 Å (Table S3) indicate near-atomic similarity of the arrangements. The spatial distribution of the clusters is shown in Figure S7.

Most of the clusters contain arrangements of one simulation only, however cluster C6 is visited by all simulations and cluster C5 is visited by all but simulations 5 and 9. Two pairs of simulations share several clusters. Simulations 7 and 8 share 4, simulations 1 and 4 share 3 clusters, demonstrating high similarity between these pairs of simulations. Even if the clustering identifies simulations 5 and 9 as outliers, i.e. no clusters except the cluster C6 are shared with any other simulation, the center of mass of HasA travels through very similar regions (see Fig. 7) and large RMSD values have to be explained by different rotational orientations of the proteins against each other (see below for more details). In contrast, a very exceptional path of the center of mass can be seen for simulation 9 (light grey spheres in Fig. 7). Due to its singularity, we consider this path as less probable and will not discuss it further. The clustering can also be used to characterize single simulations. Each simulation is represented by a continuous string of clusters from the starting arrangement to the end structure along the target RMSD axis. The arrangements of the last 10 ns of simulation 1 (target RMSD between 10 and 0 Å), e.g. were assigned to five clusters interpretable as four intermediates plus the final state of the simulated approach. Taking all these information together, a tree-like graph of the clusters can be drawn connecting consecutive clusters and, in this way, highlighting the number of intermediates per simulation, as well as similarities shared by different simulations (Fig. 8). The exceptional path of simulation 5 can be seen here as well as from Fig. 7.

### Comparison of simulated arrangements with the DM complex

**Fig. 9:**
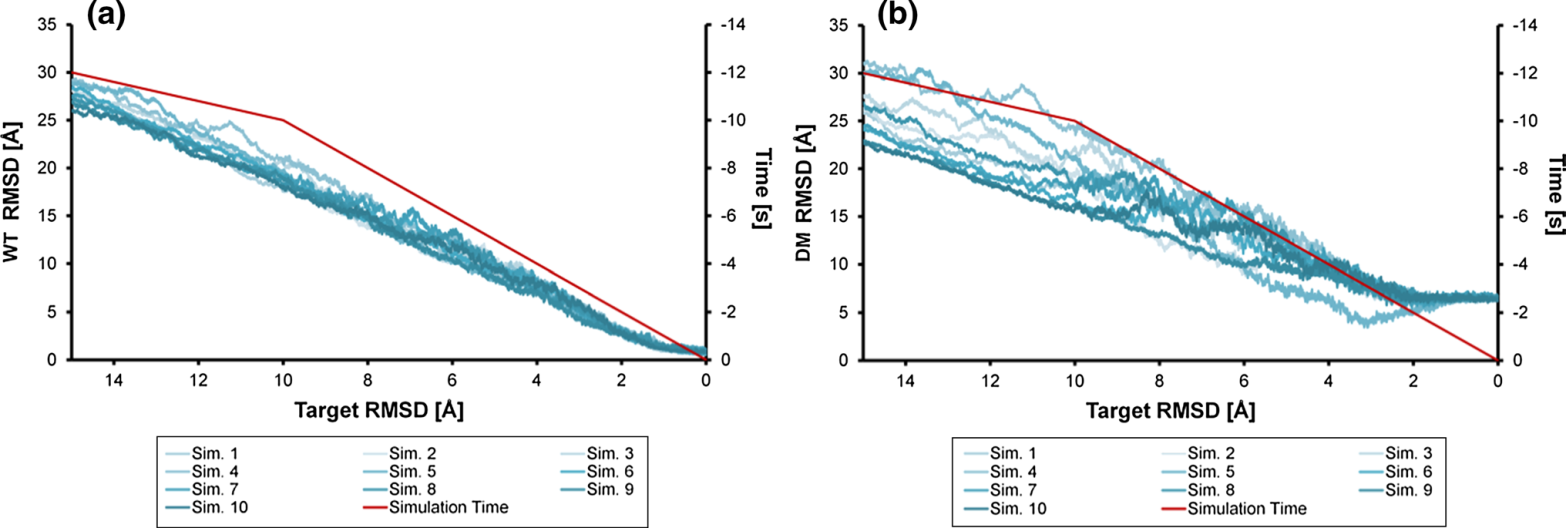
Target RMSD plot of the C_α_ RMSD of holoHasA and HasR arrangements during the ten independent targeted MD simulations with respect to the WT complex (WT RMSD, **a**) and DM complex (DM RMSD, **b**), respectively. Red: target RMSD vs. simulation time highlighting the switch from the fast to the slow reducing rate. Depending on the starting arrangement different paths are followed, which finally converge towards the WT complex. Since the simulation time does not correspond to the real time and, thus, has no physical meaning due to the artificial target potential, we use here and in the following the target RMSD to specify specific points during the simulation

RMSD values for all holoHasA and HasR C_α_ atoms of the simulated arrangements to the corresponding atoms in the WT complex and the DM complex were calculated, in the following called WT RMSD and DM RMSD, respectively. The arrangements were not aligned before the RMSD calculations, since they already have the same HasR-based reference coordinate system due to the position restraining of the β-barrel. The period with target RMSDs between 15 and 0 Å is shown in Fig. 9. These WT and DM RMSD values fluctuate much more than the current RMSD,since atoms are included which are not constrained by the target potential. The DM RMSD values (Fig. 9b) again reflect the already discussed variety of possible approaches. Notably, after the target RMSD value falls below 4 Å, all simulations except no. 5 behave very similar regarding their DM RMSD.

To understand the specific behavior of simulation 5, we will compare it with simulation 7 as a representative for the other simulations. Two movies of these (sim5.mpg and sim7.mpg) are provided in the Supporting Material.

**Fig. 10:**
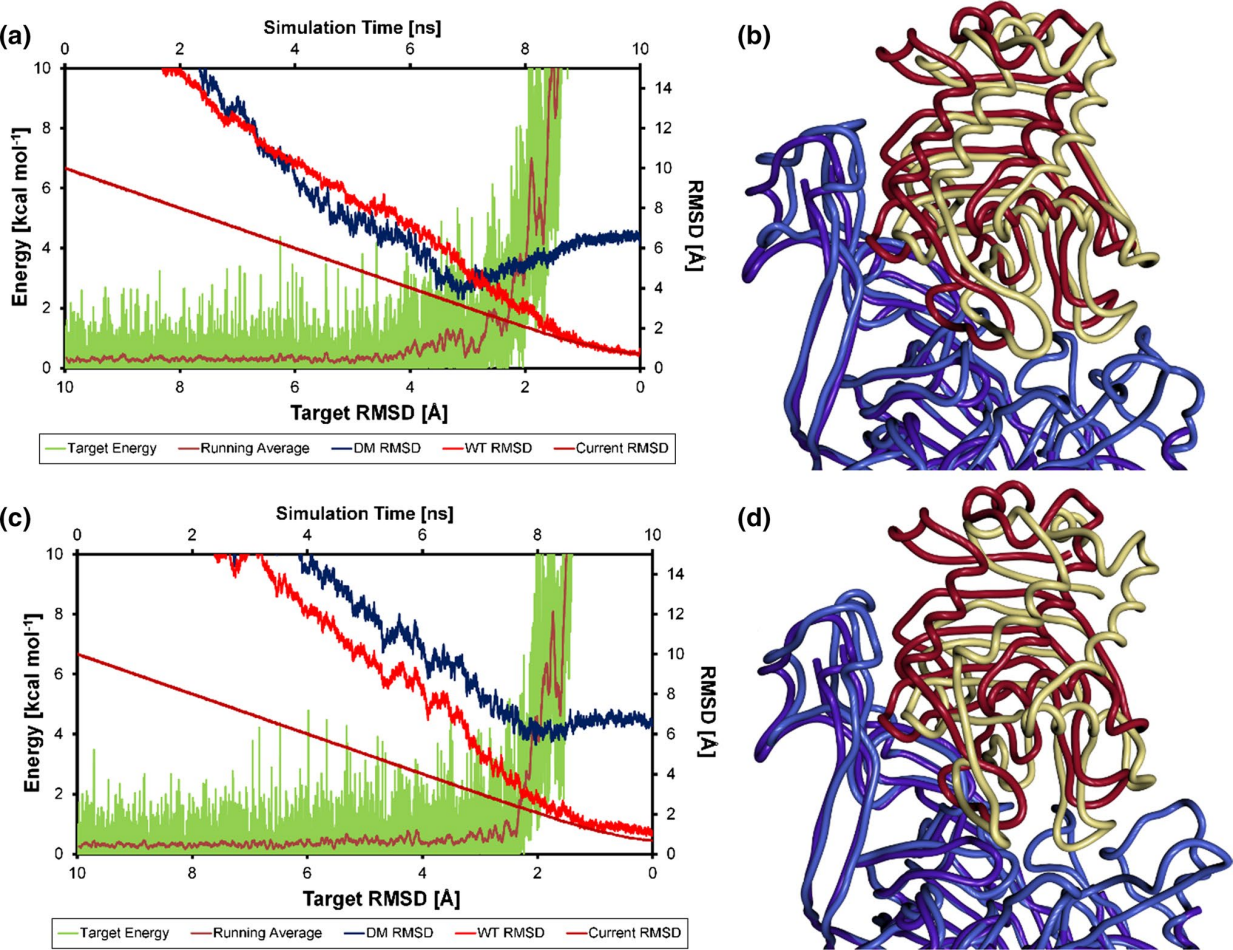
**a** Target RMSD plot of simulation 5. Red: WT RMSD; blue: DM RMSD, green: target potential (running average) given by the energy scale at the left; brown: current RMSD. Values are running averages over 25 arrangements before and after the specified target RMSD. **b** The DM-like arrangement from simulation 5 at a target RMSD of 3.1 Å yielding the lowest DM RMSD of 3.45 Å (light blue HasR and green holoHasA,) superimposed with the DM complex (dark blue HasR and red holoHasA). **c** Target RMSD plot of simulation 7. Red: WT RMSD; blue: DM RMSD, green: target potential (running average) given by the energy scale at the left; brown: current RMSD. Values are running averages over 25 arrangements before and after the specified target RMSD. **d** The arrangement from simulation 7 at a target RMSD of 2 Å yielding the lowest DM RMSD (light blue HasR and green holoHasA) superimposed with the DM complex (dark blue HasR and red holoHasA). Compared to the DM complex, holoHasA is rotated here towards CA2 resulting in a large DM RMSD. Nevertheless, the lower parts of CA1 (see Fig. 1) are addressed quite similarly in simulation 7 and the DM complex. The tilt towards CA2 can be explained by interactions with loops L2 and L3 of CA2. These interactions are absent in the DM complex due to the higher flexibility introduced by the point mutations resulting in a destruction of CA2 contacts (see Supporting Material, MD simulations of WT-HasR alone and DM-HasR alone)

The DM RMSD is larger than 5 Å in all simulations except in simulation no. 5 (Fig. 9b), where it drops transiently down below 4 Å. In the latter, a minimal DM RMSD of 3.45 Å is observed at a target RMSD of around 3.1 Å. This unique arrangement is referred to as DM-like in the following. In comparison, the WT RMSD is still 5.35 Å at that time. Based on the DM RMSD, we can conclude that simulation 5 passed through an intermediate DM-like arrangement, which is more similar to the DM complex than any arrangement of, e.g., simulation 7 (compare Fig. 10b, d), but is still clearly different from the DM complex on an atomic length scale. As seen in Fig. 10b, the main differences of this DM-like arrangement compared to the DM complex are a shift to the right and rearrangements in loops L6 and L9 (CA1) of HasR to compensate for this move.

To compare the simulations 5 and 7 energetically, the current RMSD and the corresponding target potential, as well as its smoothed running average are represented in Fig. 10a, c. In both simulations, the current RMSD follows the target RMSD almost perfectly until the latter reaches 4 Å. This translates to a maximum target potential of less than 6 kcal/mol everywhere before this point. Thus, there are no major potential energy barriers, which have to be overcome in this phase of approach of holoHasA toward HasR and the target potential is only needed to bias the relative motion of the two molecules toward each other. However, the final approach at target RMSD values below 4 Å is then associated with larger energy fluctuations and a general increase of the target potential. At this time, specific interactions of the WT complex are beginning to form, which apparently require breaking intermediate, but nevertheless strong interactions. One such event can be identified in simulation 5, when the DM-like structure has formed. The target potential shows a clear increase and then a drop at 3 Å target RMSD to almost the values seen in the first half of the simulation (Fig. 10a). This reflects an energy barrier and shows that there is a true metastable intermediate at this RMSD. This transforms further into a cluster no. 5 arrangement, which follows at a target RMSD of 2.85 Å.

Another way to follow the course of complex formation in the two simulations is the percentage of native contacts shared with the WT complex and the DM complex (see Pairwise contacts in Supporting Material). In both simulations, the number of native contacts shared with the DM complex reaches a plateau of about 60% at a target RMSD of about 2 Å, whereas those shared with the WT complex increase further to a value of about 85% when the target RMSD has approached zero (see Figure S8).

The different paths of approach to the two contact areas are reflected by the displacement of water molecules in the course of complex formation (see Supporting Material). In simulation 5, desolvation of CA1 lags behind CA2 down to a target RMSD value of about 2 Å, whereas in simulation 7 both contact areas are simultaneously dehydrated (see Figure S9 a, b).

## Discussion

### Structural and functional data

We had previously shown that protein–protein interaction drives heme transfer from the HasA hemophore to its HasR receptor in a highly efficient process allowing heme uptake by the bacterium at very low extracellular heme concentrations (Izadi-Pruneyre et al. 2006). The structure of the WT complex together with the structure of the Ile671Gly complex (Krieg et al. 2009) hinted at the late steps of the heme transfer reaction, once holoHasA has come in close contact with HasR. The structures show the presence of two contact areas on the receptor, referenced here as CA1 (loops 6, 8 and 9) and CA2 (loops 2, 3, 10 and 11), contacting the β-face of HasA and the A2 loop of HasA, respectively. The A1 heme-binding loop of HasA is in both crystal structures invisible in the electron density and the visible chain termini show that it has undergone a large pivoting movement. While the heme is relocated to the HasR-binding site in the WT complex structure, it is still found bound to the HasA A2 loop in the Ile671Gly complex structure. We hypothesized that for an efficient heme transfer to occur, a strong interaction was needed requiring burying of the contact areas to allow for a specific, correct positioning of the HasR Ile671 side chain poised for the clash with the heme-binding loop A2 in the HasA heme-binding pocket.

The focus of this study was on the initial steps of HasA–HasR binding, which would lead to this specific arrangement. On the basis of inspection of the WT complex, we constructed the DM-HasR double mutant in CA2, which is able to bind holoHasA, but not to transfer heme. In the DM complex, the CA2 area was strongly affected by the mutation and most of the respective loops were not seen in the electron density map indicating a static or dynamic superposition of different conformations in the crystal. Moreover, the HasA contacts with CA1 were different from those in the WT complex, except for one H-bond. Furthermore both heme-binding HasA loops were in the same conformation as in holoHasA. An equivalent “mirror” mutant in CA1 could not be obtained. In contrast, in the Ile671Gly complex structure, we find HasA in its final position, i.e. both CA1 and CA2 contacts formed and loop A1 from HasA invisible as in the WT complex, although heme is still on HasA.

This prompted us to the working hypothesis that the DM complex corresponds to a transient "encounter complex" preceding the Ile671Gly complex and the WT complex suggesting that complex formation is an ordered process with CA1 being first contacted by HasA. It also hinted that contacts with CA2 and sliding of HasA along CA1 might be necessary to achieve the release of loop A1 from HasA. This view is in accord with the thermodynamic parameters of the interaction of holoHasA with DM-HasR and with the Ile671Gly-HasR mutant (Table 2).

### Molecular dynamics

The free dynamic simulations of the DM complex structure and the same structure without the mutations were in accord with the behavior of the proteins in solution. In the system with the DM mutant, the two proteins show no tendency to dissociate. However, in the system with the WT-HasR, a majority of the simulations develops toward the WT complex. How is it possible that the potential surface of the WT system in a DM complex structure, which is sufficiently downward sloping so that thermal energy quickly overcomes local minima on its path to the WT complex is changed by two relatively remote mutations in CA2, Arg297Ala and Asn800Ala, so that a deep potential minimum is created. One candidate for such a far-reaching effect is the loss of the positive charge on Arg297. Another explanation is that missing interactions of HasA with CA2 in the DM mutant could suspend an induced fit effect allosterically transduced through HasA (Changeux 2011) by which WT-HasR levels out the potential minimum of the CA1 interactions.

While these results support the suspicion that the mutations create an unphysiologic potential minimum into which the proteins are trapped, they do not rule out that the physiologic system might visit this arrangement of holoHasA and HasR as a necessary intermediate during complex formation.

We addressed this latter question by targeted molecular dynamics simulations of the binding process. As already discussed in the results section, the initial approaches of the two proteins follow different paths in the simulations which were started at different positions of unbound holoHasA relative to HasR. The convergence of all simulations to cluster no. 6 could be questioned, because it is biased by the target potential. Yet this is a well-justified constraint, because we know that in vivo the HasA–HasR arrangements must converge to the WT complex, which leads to heme transfer. The other question with respect to the targeted MD is whether the simulations follow the same low-energy paths as the native binding processes. The low target potentials during most of the simulation period, never exceeding 4 kcal/mol, the clustering of the arrangements and the fact that some of the late clusters are visited by several simulations are in our opinion good evidences that the simulations reproduce native events, but obviously this is no proof. Yet, none of the simulations passes through the DM complex, only one passes nearby at a lowest DM RMSD of 3.45 Å. This is at odds with the hypothesis that the DM complex is a common intermediate of all binding processes, although due to the limitations of the MD approach they should also be interpreted with caution. It is possible that without the MD abstractions, e.g. absence of the target potential and presence of an LPS outer membrane leaflet, a binding path visits the DM complex instead of passing nearby or even that a path starting with contacts at CA2 becomes less favorable Nevertheless, the general finding of the simulations that several initial contacts of HasA with HasR are possible and likewise several paths of diffusion on the HasR surface toward the WT structure can occur, seems reasonable, because it means that the entropy loss of binding occurs in a more continuous manner as in the case of the working hypothesis drawn from the DM structure.

If we trust the general features of the MD simulation, the binding of HasA to HasR can be subdivided into three phases. Free diffusion as long as both proteins have not yet formed contacts at target RMSDs > 10 Å, sliding of holoHasA on the external surface of HasR at target RMSD values between 10 and 2 Å and the late steps comprising the conformational changes leading to heme transfer.

As to the first phase, the mutual diffusion will be biased by the attractive electrostatic potential. When forming first contacts, the long positively charged loops of HasR, particularly, loops L6 and L9 from CA1 and loops L2 and L3 from CA2 seem to function like two tentacles. Which one of the two areas is addressed first by HasA depended on the starting position of HasA.

Simulations 5 and 7 were presented in more detail in the results section as representative examples. Two movies of simulations are provided in the Supporting Material. In simulation no. 7, HasA is first bound at the tip of loops L6 and L9 (CA1). After some reorientations involving larger rotations in all directions, the protein is then pulled toward the other tentacle and forms the first contacts with CA2. In contrast, in simulation no. 5, first contacts are formed with CA2. These appear at a target RMSD of 10 Å and differ completely from those seen in the WT complex. Even taking all ten simulations into account, we cannot deduce which of the two approaches will occur more frequently under native conditions, although common sense would hint to CA1 with its long loops, which are presumably less masked by the LPS layer than the CA2 loops. The restrictive conditions of targeted MD cannot be taken as a framework for a representative sample of the native distribution of trajectories.

The cluster analysis suggests that in the course of the simulations, well-defined intermediate binding arrangements are visited and that the simulation paths following different initial arrangements gradually coalesce into a single arrangement at a target RMSD of 1.25 Å.

The diffusion process of HasA on the surface of HasR reminds of a greasy slide (Schirmer et al. 1995; Meyer and Schulz 1997; Forst et al. 1998), where HasA is temporarily connected to the tentacles and exploits alternative binding modes. This process is biased or funneled to reach the common final arrangement at a target RMSD below 2 Å.

In the late steps, a fast increase of contacts shared with the WT complex is observed (Figure S8). In simulation 5, this begins directly after passing the DM-like arrangement and can, thus, be assigned to the transition from the DM-like arrangement to the final WT complex.

In this range of low target RMSD values, we observe a strong increase in the target potential beyond 10 kcal/mol for all simulations. Immediately before, all simulations have converged to the cluster 6 arrangement (at target RMSD 1.25 Å), which shares 75% of interactions with the WT complex. HasA is still in its holo conformation with heme coordinated by both loops A1 and A2, but during the phase of steep increase of the target potential, loop A1 shows the strongest conformational changes seen in HasA. This suggests that from the cluster 6 arrangements the critical conformational changes effecting the dissociation of the heme will develop. Obviously, well defined and specific conformational changes involving loop A1 and later loop A2 of HasA, as well as loops of HasR have to take place which require the passage of potential energy barriers. The specificity of these changes requires time-consuming sampling of the conformational space, i.e. low rate constants, so that they proceed on a much longer timescale than the ns scale of the sliding motion of HasA on HasR. The enforced constant decrease of the target RMSD does not grant sufficient time to the system, which resists it as seen from the target potential. In a targeted MD, Salo-Ahen and Wade (Salo-Ahen and Wade 2011) observed similarly steep restraint energy changes to more than 100 kcal/mol upon conformational changes associated with dihedral angle alterations.

Although the formation of the complex and heme transfer together are very exergonic (Létoffé et al. 2001), they proceed at low rate which hints to the inability to simulate the late steps.

At still smaller target RMSD values, the force resulting from the target potential will drive the system artificially toward the unique crystal structure, which is the mean of the statistical ensemble of structures that are actually present in the crystal (Burnley et al. 2012; Kuzmanic et al. 2014). To better reflect the real process, the MD should drive the system toward a thermodynamic ensemble of complex structures. This artifact of the simulation will also contribute to enlarged target potentials.

Due to these considerations, the simulation at target RMSD values below 1.8 Å must be considered as unreliable.

## Conclusion

The combination of a new mutant X-ray structure showing a putative intermediate of the binding process and molecular dynamic simulations based on the new and the wild-type structures stimulated us to propose consistent sequences of events for HasA–HasR complex formation coupled with heme transfer. Independent of the starting arrangement in the simulations, holoHasA is captured by far-reaching electrostatic interactions and guided via changing contact interactions into a well-defined bound arrangement sharing 75% of the WT complex interactions. While this is a fast process, the subsequent endergonic heme deconjugation from HasA by removal of the loops A1 and A2 requires this specific and precise arrangement and proceeds on a much longer timescale inaccessible to the MD simulation used here. Heme deconjugation should take place within a tight complex of both proteins to prevent heme loss during transfer to HasR. The DM complex shows the contributions of CA1 and CA2 for binding of HasA. The molecular dynamics results suggest that this arrangement of the two proteins is not a necessary intermediate for forming the WT complex.

## Electronic supplementary material

Below is the link to the electronic supplementary material.

Supplementary file1 (PDF 4355 kb)

Supplementary file2 (MPG 31484 kb)

Supplementary file3 (MPG 29756 kb)

## Abbreviations

apoHasA: HasA without bound heme
CA1: Contact area 1
CA2: Contact area 2
DM complex or DM complex structure: holoHasA~DM-HasR crystal structure
DM-HasR: Double mutant HasR (Arg297Ala/Asn800Ala)
DM-like arrangement: Arrangement in simulation 5 at target RMSD of 3.1 Å
Ile671Gly-HasR: Single mutant HasR (Ile671Gly)
Ile671Gly complex: holoHasA~HasR (Ile671Gly) crystal structure
holoHasA: HasA complexed with heme
ITC: Isothermal titration calorimetry
MD: Molecular dynamics
RMSD: Root mean square deviation
Current RMSD: RMSD between a HasA–HasR arrangement and the WT complex for the target mask atoms
DM RMSD: RMSD between a HasA–HasR arrangement and the DM complex for all C_α_ atoms
Target RMSD: RMSD value set as target in the MD target potential
WT RMSD: RMSD between a HasA–HasR arrangement and the WT complex for all C_α_ atoms
C_α_ RMSD of holoHasA to the WT complex: RMSD between the C_α_ atoms of holoHasA in a HasA–HasR arrangement and in the WT complex
C_α_ RMSD of holoHasA to the DM complex: RMSD between the C_α_ atoms of holoHasA in a HasA–HasR arrangement and in the DM complex
Short simulations: Simulation runs during a 6 ns period
WT complex or WT complex structure: apoHasA~HasR~heme crystal structure
WT: Wild-type HasR
