# Supplementary material for: Binding of HasA by its transmembrane receptor HasR follows a conformational funnel mechanism
Source: Eur Biophys J. 2019 Dec 4;49(1):39–57. doi: 10.1007/s00249-019-01411-1 (PMC6981324; doi:10.1007/s00249-019-01411-1)
Supplement: Supplementary file 1 — Supplementary file1 (PDF 4355 kb) [file 249_2019_1411_MOESM1_ESM.pdf]

Supporting Material:

Two movies of simulations 5 (sim5.mpg) and 7 (sim7.mpg) are provided. The targeted simulation of HasA and HasR molecules towards the static structure of the WT complex is shown. The yellow tubes represent the backbone of the proteins. The heme group is shown as a CPK model using conventional colour code.

Table S1

HasA	distance [Å]	HasR	loop
H-bonds			
CA1			
ASN 62 (O)	2.57	SER 547 (OG)	L6
ASN 41 (OD1)	3.32	SER 670 (OG)	L8
GLY 43 (O)	3.00	ILE 671 (N)	L8
SER 58 (O)	3.78	GLN 668 (NE2)	L8
SER 59 (O)	3.12	GLN 668 (NE2)	L8
GLN 63 (OE1)	2.82	GLN 668 (NE2)	L8
ASP 8 (OD1)	3.87	ARG 732 (NH2)	L9
SER 49 (OG)	2.79	GLY 752 (O)	L9
GLN 109 (NE2)	3.81	GLY 730 (O)	L9
GLN 109 (NE2)	3.77	GLY 728 (O)	L9
ASP 102 (OD2)	2.71	SER 744 (OG)	L9
PRO 111 (O)	3.74	ARG 732 (NH2)	L9
CA2			
ASN 79 (ND2)	3.01	ASN 300 (OD1)	L2
PHE 78 (O)	3.34	ARG 297 (NH1)	L2
GLU 80 (O)	3.62	THR 302 (OG1)	L2
GLU 80 (OE1)	3.56	THR 302 (OG1)	L2
GLU 80 (OE2)	3.17	TYR 308 (OH)	L2
GLN 124 (NE2)	3.50	ASN 300 (OD1)	L2
GLN 126 (O)	3.85	TYR 367 (OH)	L3
ALA 82 (N)	3.63	ASN 360 (OD1)	L3
LEU 50 (O)	3.87	ASN 800 (ND2)	L10
TYR 75 (O)	3.40	ASN 800 (ND2)	L10

SER 42 (OG)	3.79	SER 605 (O)	L7
Salt bridge			
ASP 8 (OD1)	3.87	ARG 732 (NH2)	L9

HasA	Distance[Å]	DM-HasR	loop
H-bond			
CA1			
ASP 96 (OD2)	3.79	LEU 543 (N)	L6
ASP 96 (OD2)	3.78	THR 544 (N)	L6
GLY 44 (N)	3.32	PRO 669(O)	L8
SER 58 (OG)	3.77	VAL 667 (O)	L8
ASP 39 (OD2)	3.41	ASN 673 (N)	L8
ALA 40 (O)	3.58	GLN 668 (NE2)	L8
CA2			
VAL 37 (O)	3.44	SER 605 (OG)	L7
ASP 39 (OD1)	3.83	SER 605 (OG)	L7
ASP 39 (OD2)	3.39	THR 607 (N)	L7
salt bridge			
ASP 102 (OD2)	3.88	LYS 718 (NZ)	L9

Table S1: List of H-bonds and a salt bridge in the interface between HasA and HasR (upper panel) as well as HasA and DM-HasR (lower panel) in the WT complex and the DM complex, respectively, assembled with PISA [Krissinel, E., and K. Henrick. 2007. Inference of macromolecular assemblies from crystalline state. J.Mol.Biol. 372:774–797.]

Table S2

CA2 mutants

R297A: GACATCGGCAACATTGCCATCAATAACGAC

R297Ar: GTCGTTATTGATGGCAATGTTGCCGATGTC

N300A: AACATTCGCATCAATGCCGACACCGGCAAT

N300Ar: ATTGCCGGTGTCGGCATTGATGCGAATGTT

Y308A: GGCAATTACGATCGCGCCGCCGAGAGCATC

Y308Ar: GATGCTCTCGGCGGCGCGATCGTAATTGCC

N800A: AGCTCGGTACCGTCCGCCTACCCGTACCTG

N800Ar: CAGGTACGGGTAGGCGGACGGTACCGAGCT

CA1 mutants

S547A: CGTCTGACCGGCTGTGCAACCACCACCCGC

S547Ar: GCGGGTGGTGGTTGCACAGCCGGTCAGACG

S744A: GAGCCTGATGCCGCGGCCAATGACTTCGTC

S744Ar: GACGAAGTCATTGGCCGCGGCATCAGGCTC

Q668A: AACCGGGGCTGGTGGCGCCGAGCATCGGCA

Q668Ar: TGCCGATGCTCGGCGCCACCAGCCCCGGTT

P669A: CCGGGGCTGGTGCAGGCGAGCATCGGCAAT

P669Ar: ATTGCCGATGCTCGCCTGCACCAGCCCCGG

S670A: GGCTGGTGCAGCCGGCCATCGGCAATGCCG

S670Ar: CGGCATTGCCGATGGCCGGCTGCACCAGCC

Table S2

Mutagenic primers :

Fig. S1

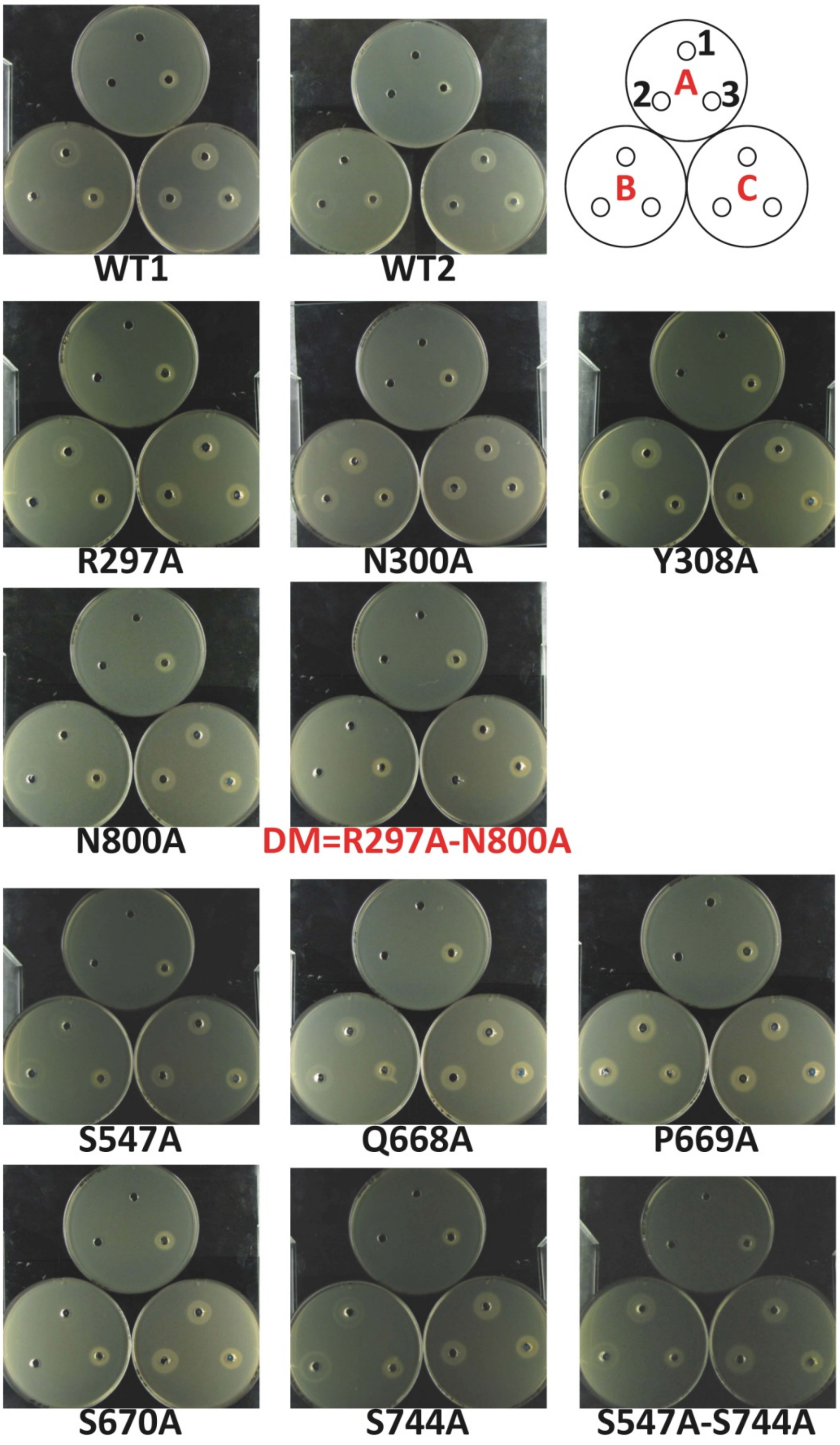

Figure S1 :

Growth tests of the *E. coli* C600Δ*hemA* strain harbouring the various recombinant plasmids encoding either WT HasR or mutants. For each strain, three plates were made (A, B, C) containing respectively 0, 50 or 150µM di-pyridyl to induce the expression of the TonB complex. Three heme sources were used, at 10µM (in 20mM Tris-HCl pH 8.0), holo-HasA

plus heme (well1), holo-HasA (well 2) and heme (well 3)). Arabinose was used at a concentration of 40µg/ml to induce HasR expression.

Further results from the growth tests:

The Pro669Ala mutant, although this residue is not involved in making a polar bond with a HasA residue, shows a distinctive phenotype: it clearly requires less TonB complex concentration to acquire heme from HasA (plate B of this mutant, wells 1 and 2, as compared to WT).

One double mutant affecting the shortest and most energetic hydrogen bonds from CA1 (Ser547Ala-Ser744Ala which form H-bonds with HasA Asn62 and Asp102, respectively) appeared to be fully functional and only slightly affected in its TonB requirements requiring less TonB complex for HasA derived heme uptake (plate B of this mutant, wells 1 and 2),

The single mutants made in CA2 showed only mild defects in HasA mediated heme acquisition, with mutant N300A and Y308A also requiring less TonB complex for HasA derived heme acquisition (again plates B for those mutants, wells 1 and 2). Mutants affected in their requirement for TonB complex are likely to be affected in steps beyond the heme transfer step between HasA and HasR.

Furthermore, as in the WT case, HasA inhibited free heme uptake by DM-HasR at low TonB complex concentrations (plate A, well 1).

Fig. S2

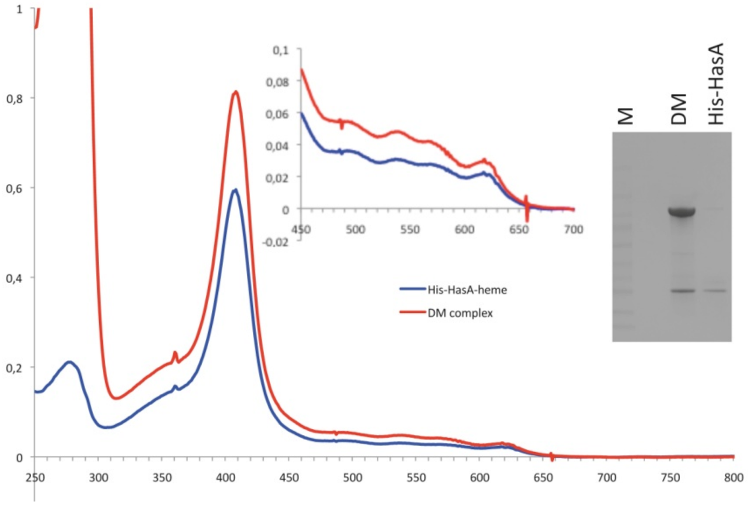

Figure S2 :

UV-visible absorption spectra of holoHasA (blue) and the holoHasA-DM complex (red)) from 250 to 800nm. The central inset shows the enlarged 450-700nm region. The rig-HasRt insert show a gel of the DM complex, and of holoHasA.

MD simulations of WT HasR alone and DM HasR alone reveal that the structure of the loops in CA2 is affected by the mutations in DM HasR

As groundwork, both the DM-HasR structure extracted from the DM complex and the WT-HasR structure extracted from the WT complex (3CSL) were analyzed independently. The DM-HasR structure was first completed by adding the missing loops in the conformation seen in the WT-HasR structure using modeller 9.10 and the side chains Arg297 (L2) and Asn800 (L10) manually replaced by alanine since the DM complex lacks six loops including the loops of the mutated residues because of missing electron density.

For better convergence, six independent simulations were performed for the WT-HasR as well as the DM-HasR using the general procedure described under “Materials and Methods”. The first had an overall time of the production run of 20 ns and the other of 10 ns. For guaranteeing a well equilibrated system the first 5 ns were removed from each simulation and 15 ns and 5 ns were used for analysis, respectively. In Figure S3 an overlay of the representative structures (snapshot closest to the average structure) of the combined ensembles is shown. It can be clearly seen that the mutations have a large influence on loop L3 (CA2) (marked with the yellow cycles). It is remarkable how this re-orientation of loop L3 influences the structure and dynamics of the complete CA2 and parts of CA1. Loop L3 (CA2) is also more flexible in DM-HasR than in WT-HasR (see Figures S4 and S5). This flexibility leads to or indicates weakened interactions between loops L3 and L4 causing a sort of domino effect finally resulting in a hinge movement of the substructure formed by L6-L9 (CA1) and an opening of the HasA binding site. These observations are in accordance with the DM complex. The more flexible loops had no clear electron density and could not be modeled.

The flexibility of the loops seen in the WT HasR alone simulations may be the reason for our inability to get crystals of the free receptor. Nevertheless, the conformations seen are sampling a relatively restricted region of the conformational space around the WT complex. Thus, the HasR structure of the WT complex is a reasonable model for the free receptor and we used it for all MD simulations described in the main publication.

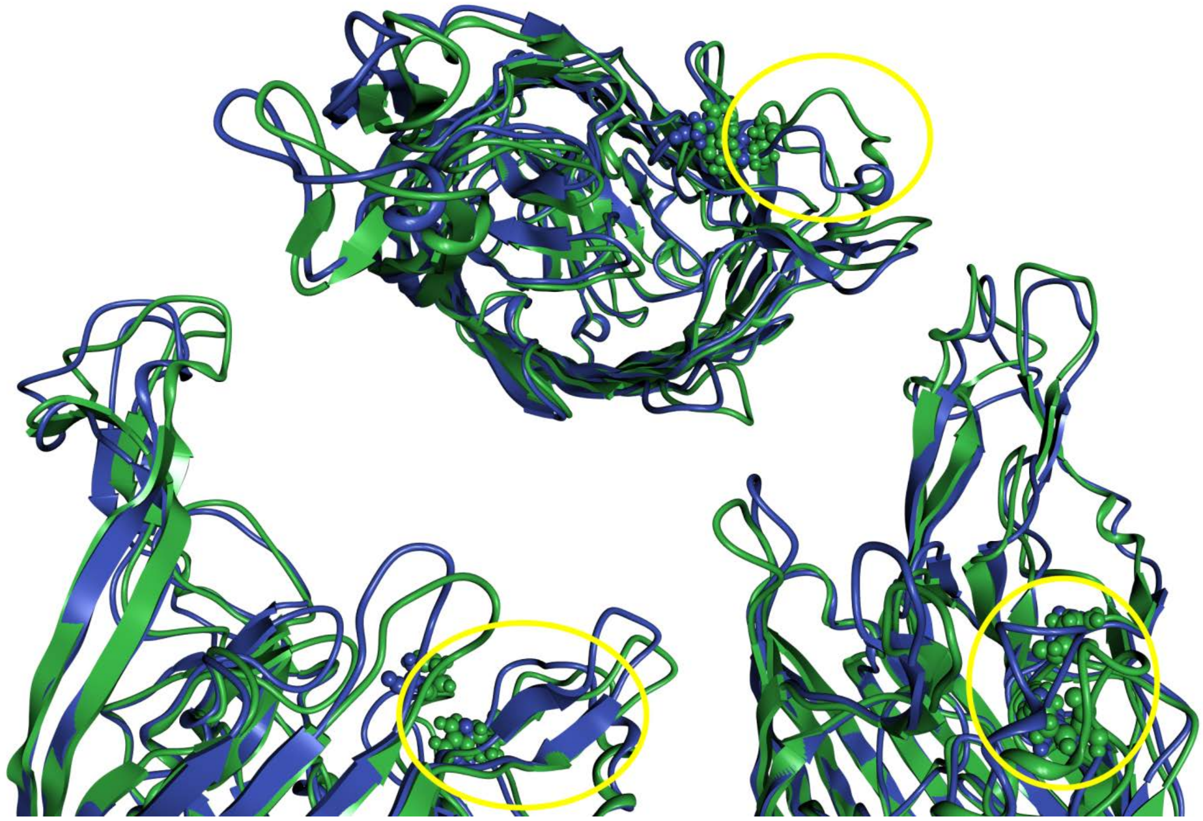

Figure S3: Top, front, and side view of the overlay of the representative simulations for DM-HasR (blue) and WT-HasR (green). The two residues mutated in DM-HasR (Arg297Ala, Asn800Ala) are shown as ball model with the same color code as the backbone representation. The yellow cycles mark loop L3, which is most strongly affected by the mutations.

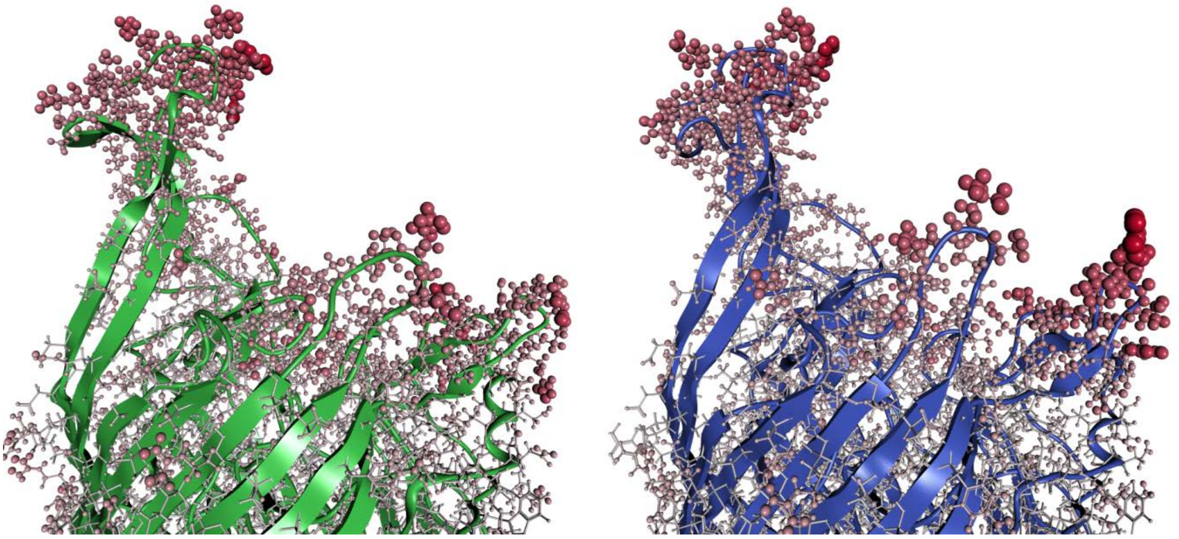

Figure S4 : Visual representation of the fluctuations in the loops of WT-HasR (left) and DM-HasR (right) in the free simulation. The root mean square fluctuations (RMSF) are encoded

as size of the atomic spheres. The increased flexibility of loop L3 can be clearly seen when comparing the right side of the figures.

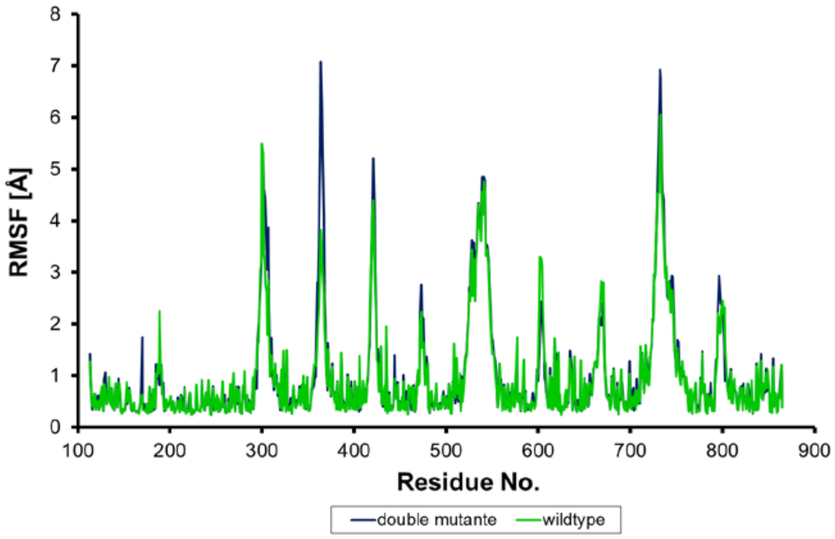

Figure S5. Per residue root mean square fluctuations (RMSF) in the free simulations of WT-HasR (green) and DM-HasR (blue). The increased flexibility of loop L3 is seen around residue 364.

In summary, both simulations (WT-HasR and DM-HasR) stay close to the structure of HasR in the WT complex with the main difference that L2 and L3 orient differently in both and the DM-HasR simulation shows a larger flexibility of these and neighboring loops. This flexibility is also found in the DM complex, where these loops are invisible. Hence for the simulations the missing HasR loops were added to the DM complex in the orientation of the WT complex structure using both the WT sequence and the DM sequence.

Paths of holoHasA upon approaching HasR

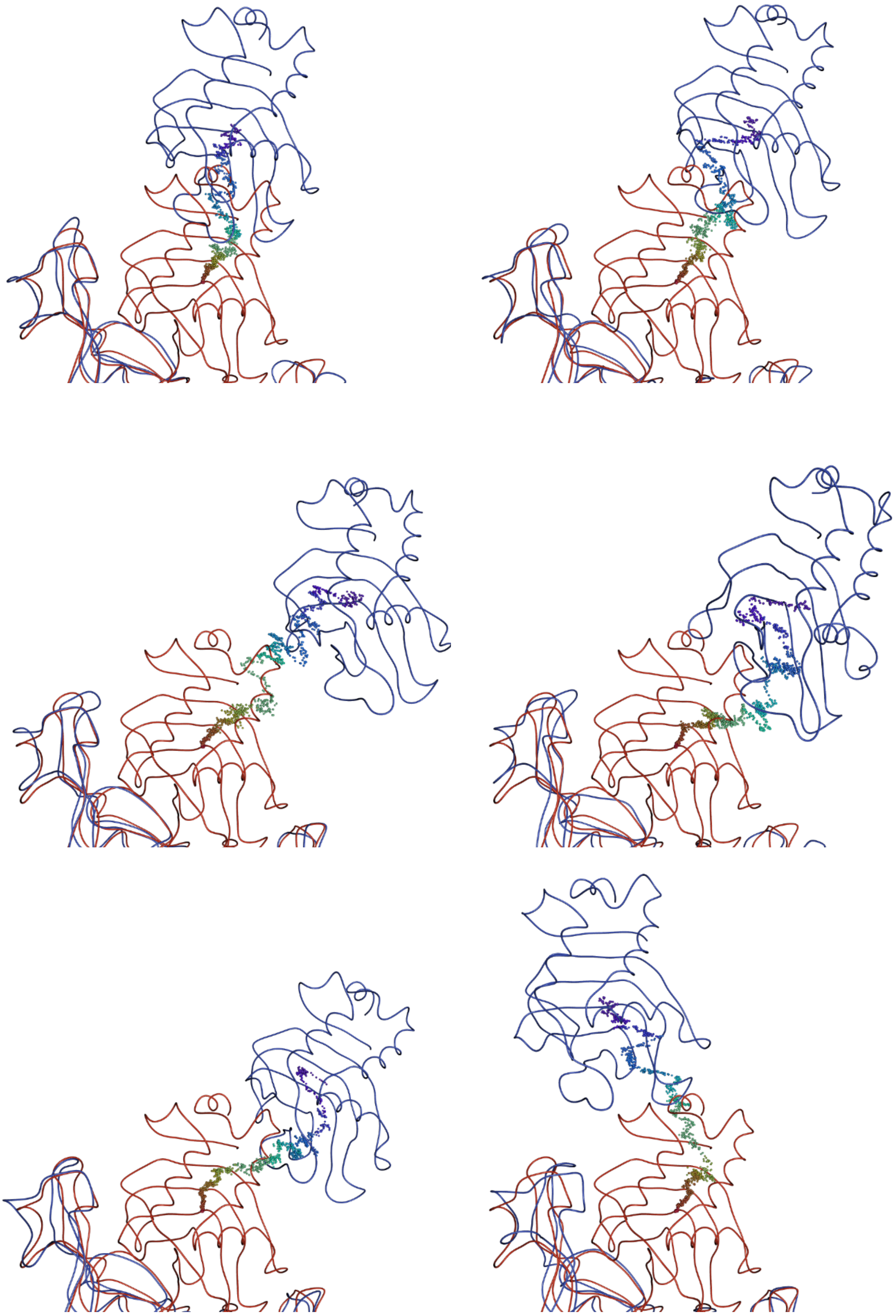

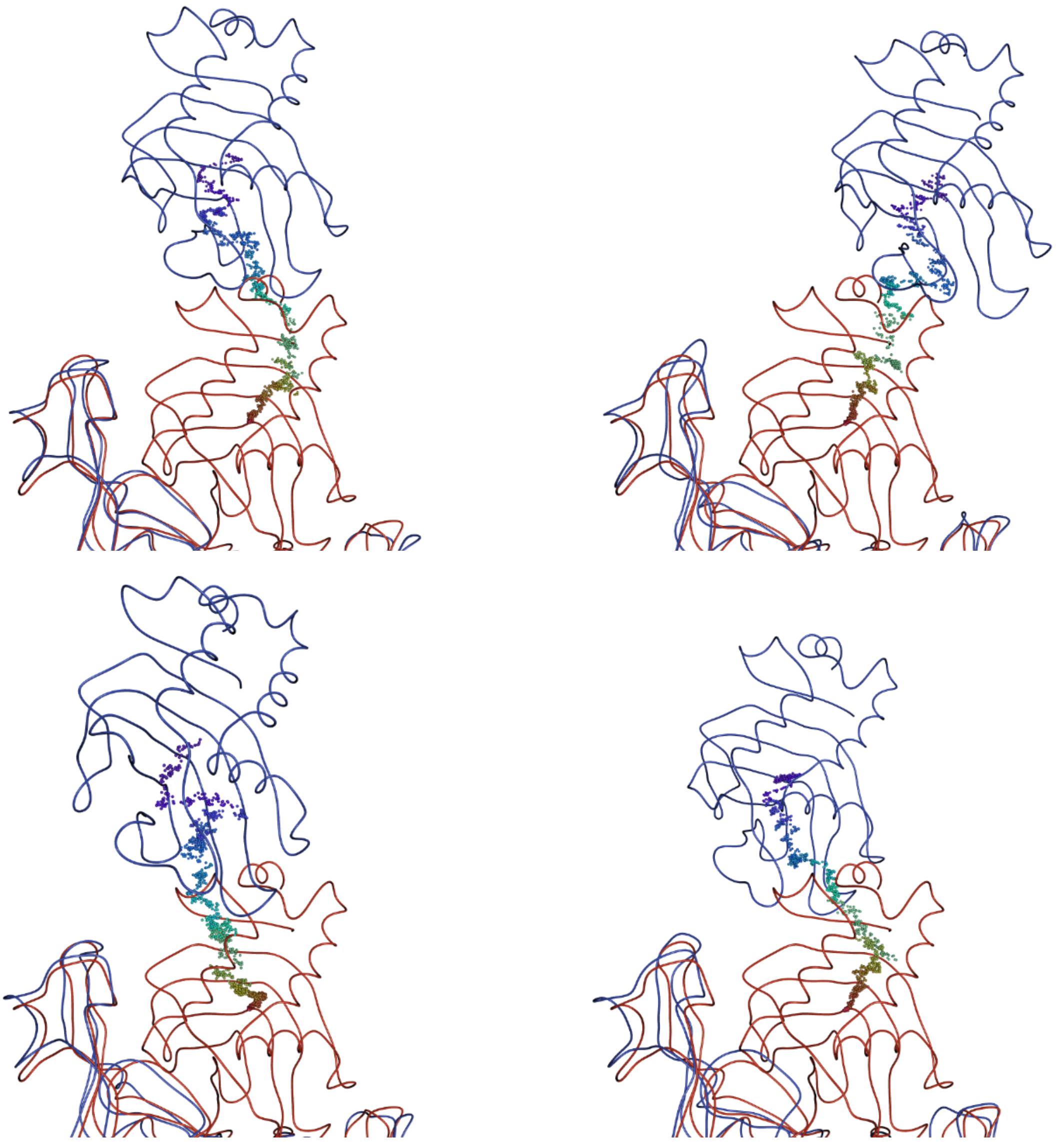

Figure S6: Spheres representing the center of mass of holoHasA during targeted MD simulation of the holoHasA-HasR complex formation for ten different starting arrangements of holoHasA versus HasR. The proteins are shown in tube representation in their starting arrangements (blue) and in the WT complex (red).

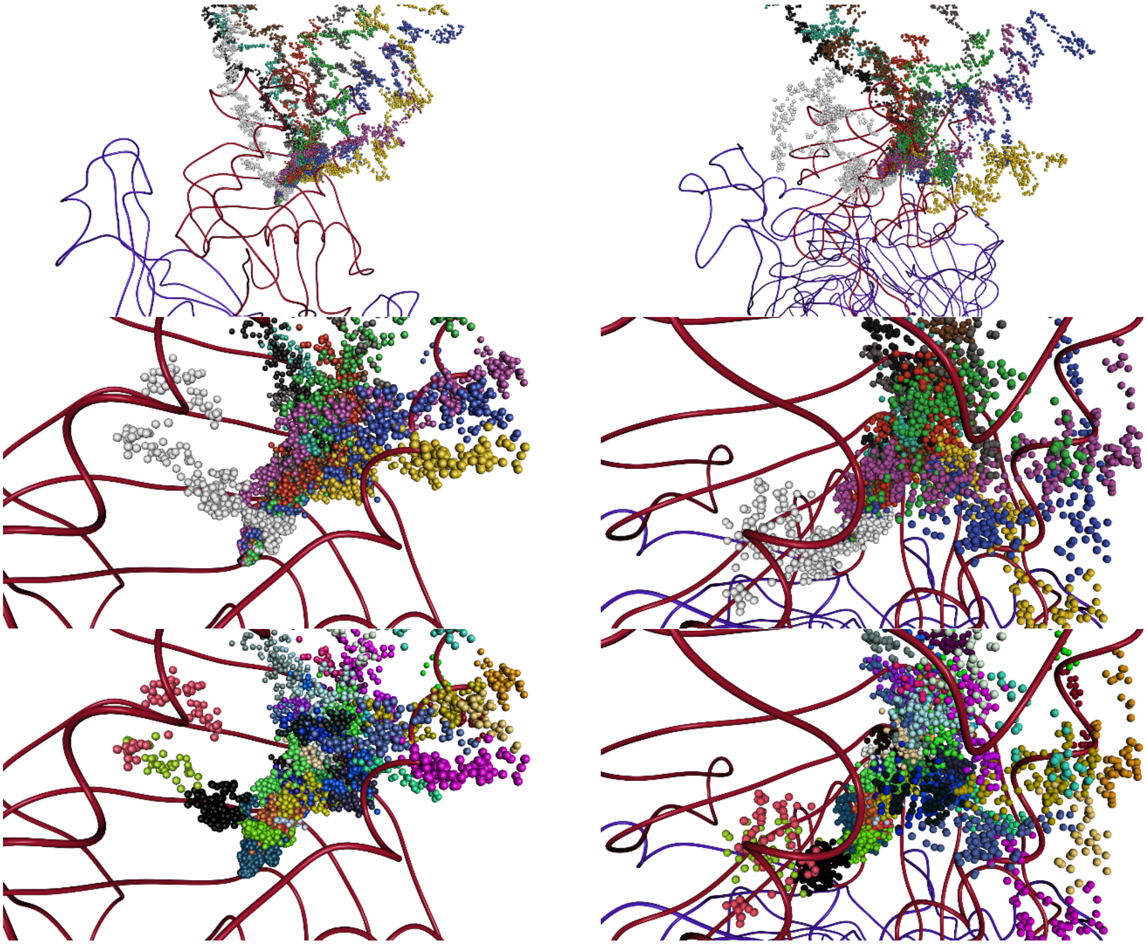

Figure S7: Cluster analysis of center-of-mass points of HasA during the ten simulations of the HasA~HasR complex formation. Left and right are two views on the binding sites from different angles. The upper part shows the overlay of the 10 paths from Figure S6 with the colors indicating different paths. A close-up of the binding site again with the colors representing different paths is shown in the middle. Finally, the lower part shows the same close-up but this time the snapshots of the paths (spheres) grouped into the same cluster are colored identically. HasA (red) and HasR (blue) are shown in tube representation.

Percentage of native contacts shared by the approaching HasA with the WT complex and the DM complex

Another way to quantify the similarity between structures is the comparison of specific interactions. To quantify these, we used the TimeScapes approach (Wriggers et al. (2009). Automated Event Detection and Activity Monitoring in Long Molecular Dynamics Simulations. J Chem Theory Comput. 5: 2595-2605) to calculate interaction patterns between protein residues defined by the distances between representative atoms of pairs of amino acids. In contrast to the original approach, we used Cα atoms as well as the carboxy carbon atoms of heme as representative atoms. If a distance is shorter than the cutoff of 7 Å at least once during the simulation (the cutoff is meant to represent an average distance between Cα atoms of two interacting residues), the time series of this distance over the MD simulation is printed in the output of TimeScapes. From these time series only those describing interactions between HasA and HasR are kept. Pairs of negatively and positively charged amino acids are classified as a salt bridge and any interaction with heme is labeled HEM. To be classified as polar, aromatic, or lipophilic, both amino acids involved have to be of this kind. The same analysis was performed for the X-ray structures of the WT and DM complex. In all simulations, numerous contacts with CA1 or CA2 have formed at a target RMSD of 10 Å (the beginning of the slow reducing rate of the target RMSD). These include salt bridges demonstrating the importance of electrostatic interactions for the early stage of the approach and for the capturing mechanism. None of these early interactions are, however, shared with the DM complex or the WT complex. According to the TimeScape approach, 120 and 65 contacts between HasA and HasR exist in the WT and the DM complex, respectively, of which 39 are shared by both. The much smaller number in the DM complex results from the impossibility to address CA2. The course of formation of contacts shared with these crystal structures during the ten simulations is shown in figure S8.

Since many contacts are common to both crystal structures the curves follow very similar trends. For simulation 5 differences are only seen at the very end after the DM-like arrangement is passed. Here, the number of contacts with respect to the WT complex further increases up to a value of 90 % while the value for the DM complex stays constant at around 60 %. For simulation 7 differences between the similarity to the WT- and DM complex are starting to emerge already early at a target RMSD of 3 Å. The contacts shared with the DM complex steeply increases at an earlier stage than in simulation 5, reaching values of 50 % to 60 % while the contacts shared with the WT complex are still around 40 %. At subsequent stages of the simulation the contacts shared with the DM complex are replaced by contacts

shared with the WT complex. At the end, the same values are reached in simulation 7 as in simulation 5 with 90 % and 60 % native WT- and DM complex contacts, respectively.

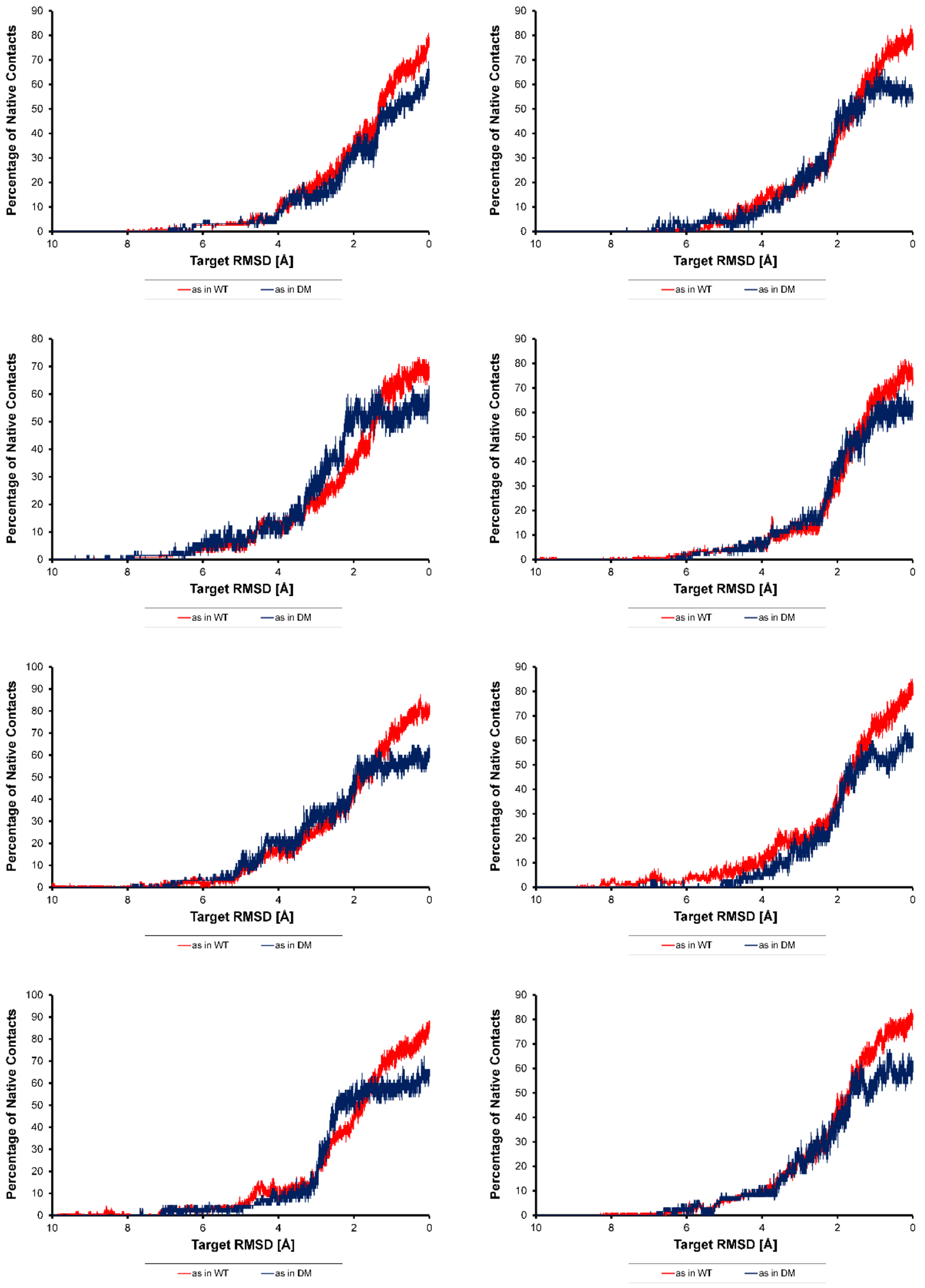

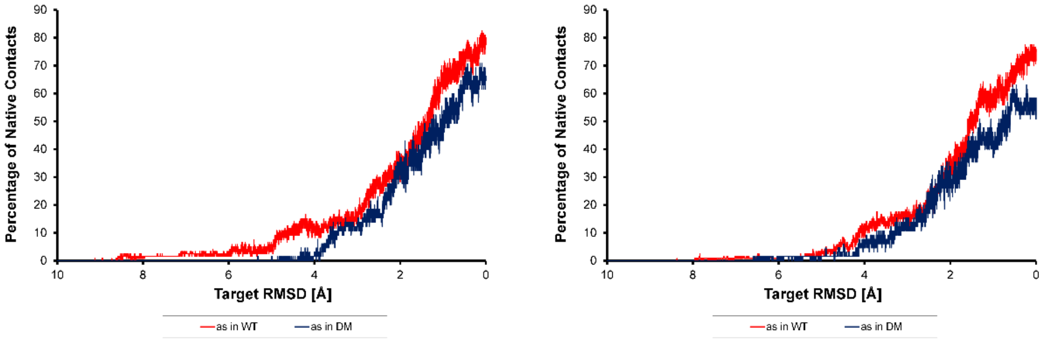

Figure S8: Target RMSD plot of the percentage of native contacts shared with the WT complex structure (red) and DM complex structures (blue) for all 10 MD simulations. The panels are arranged in horizontal numbering sequence.

Water Shells

Solvent shells around the amino acids of contact area 1 (CA1) and 2 (CA2) were calculated using the ptraj tool of the Amber10 program package (Case et al. (2008) AMBER 10. University of California, San Francisco) for all targeted MD simulations. Additionally, the solvent shells around the contact areas in a short simulation of the WT complex (using the protocol described above) were calculated. The listed values in the graphs are $(n_{first} + n_{second}) - (n_{av., first} + n_{av.,second})$, with n_{first} and n_{second} are the number of water molecules in the first and second solvent shell in the targeted MD simulation, respectively. $n_{av.,first}$ and $n_{av.,second}$ are the corresponding values averaged over the simulation of the WT complex.

Solvent shells around the amino acids of contact area 1 (CA1) and 2 (CA2)

As a final indicator for the approach of HasA towards HasR, we determined the number of water molecules close to the two contact areas (see Figures S9 a and b). In simulation 5, CA2 is already partly desolvated at a target RMSD of 10 Å. CA1 is highly solvated until shortly before the DM like arrangement where the lowest RMSD to the DM complex is reached. Then the remaining water molecules are simultaneously removed from both areas. In contrast, in simulation 7 CA1 is much earlier dehydrated even before 10 Å target RMSD. At the target RMSD with the largest number of contacts shared with the DM complex (target RMSD of 2.5 Å), CA1 is almost completely dehydrated but some water molecules diffuse in again to allow for the rearrangements needed to reach the final structure. This suggests two different possible courses of complex formation. In simulation 5 HasA initially contacts CA2 and then slides towards CA1, whereas a reversed course is used in simulation 7. Both simulations have in common that dehydration at CA2 has not been completed at the end of the simulation. There are still around 20 water molecules more in this region compared to the simulation starting directly from the WT complex. Some of these waters are located in the heme binding site of HasR and thus will be displaced only when heme approaches its final position.

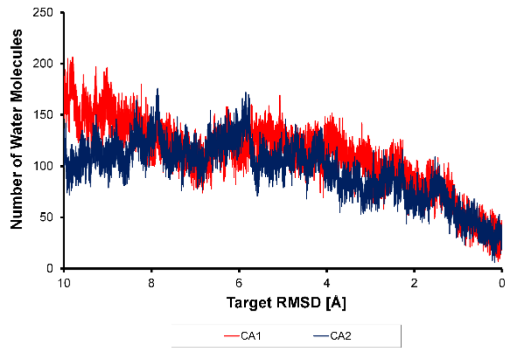

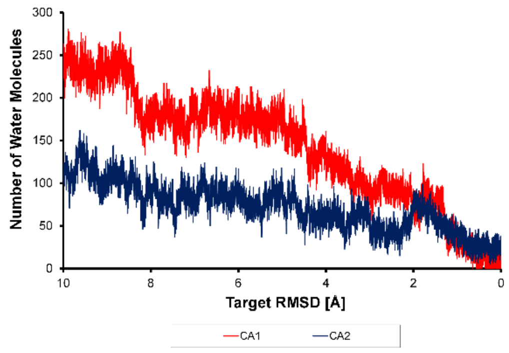

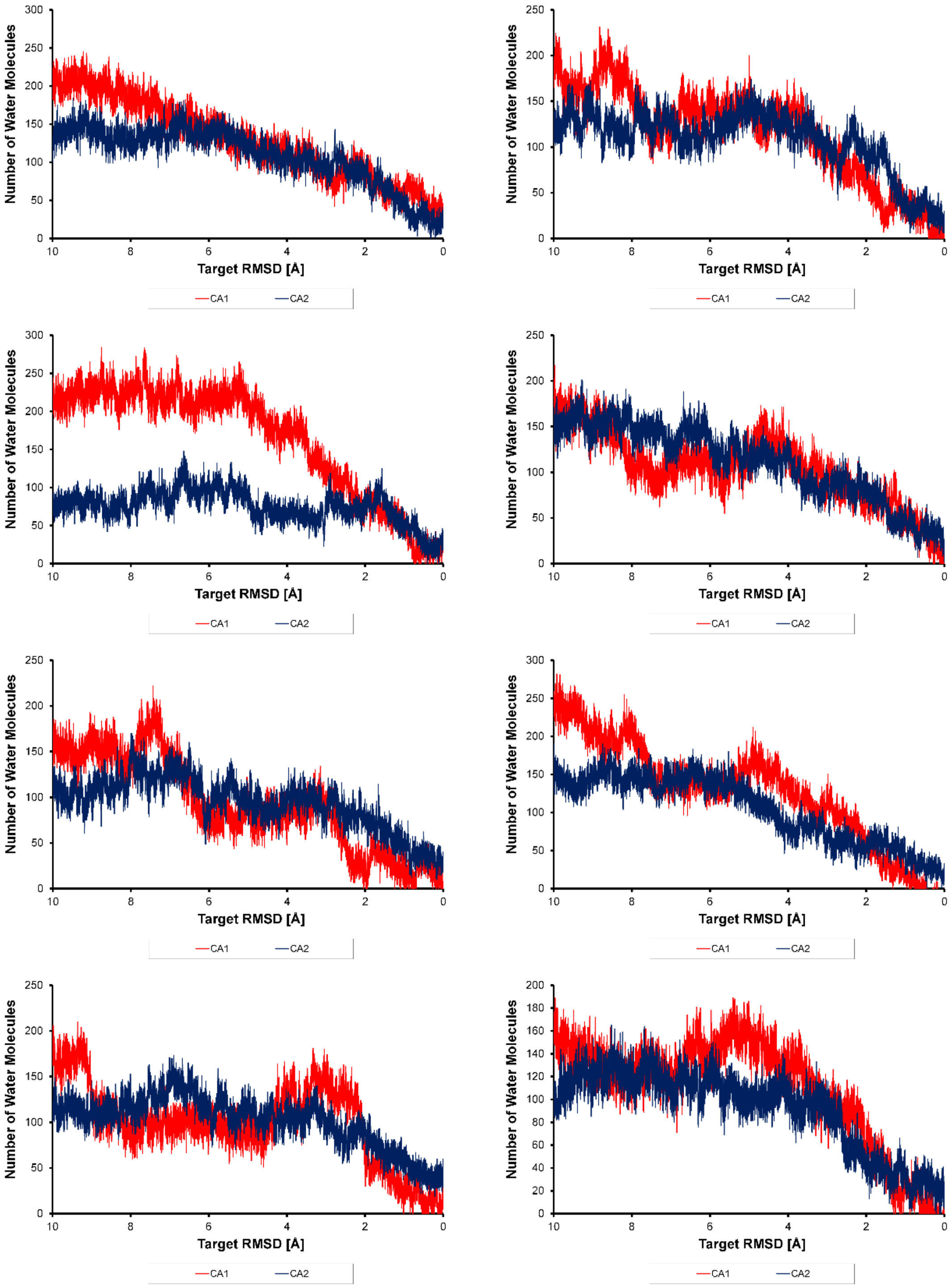

Figure S9 (a): Target RMSD plot of the number of water molecules exceeding the ones seen in the simulation of the WT complex structure close to CA1(red) and CA2 (blue) for all 10 MD simulations are shown. The panels are arranged in horizontal numbering sequence.
In simulation 5 HasA contacts to CA2 form first and contacts to CA1 follow later, in simulation 7 it is the other way round.

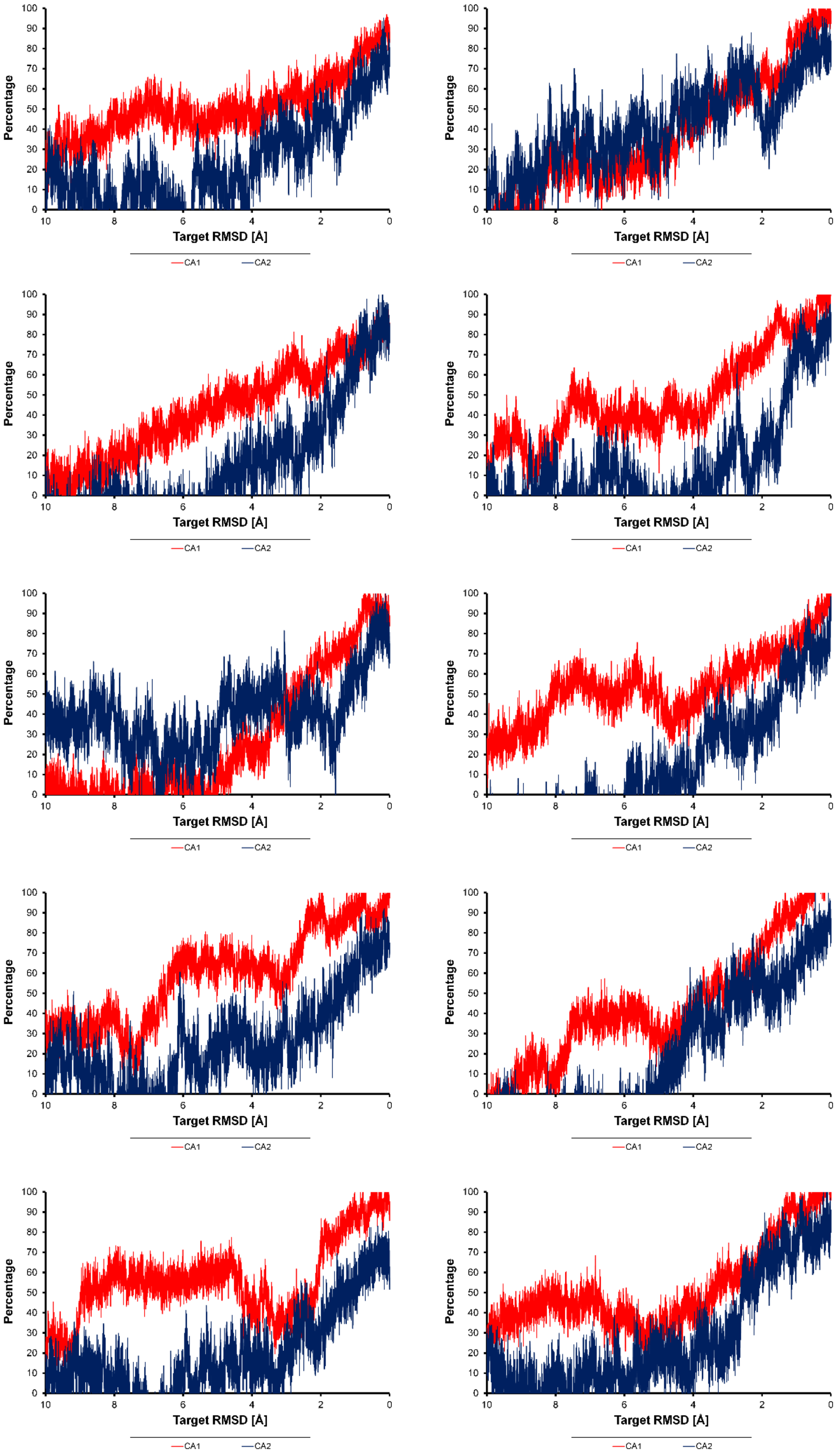
Percentage
Target RMSD [Å]
CA1
CA2

Figure S9 (b): Target RMSD plot of the percentage of replaced water molecules close to CA1 and CA2. Data for the WT- (red) and DM (blue) complex structures for all 10 MD simulations. The panels are arranged in horizontal numbering sequence.

Table S3

Cluster No.	Number of Structures	Average RMSD	Occurence	Simulations	Average Target RMSD
**1**	17	0.626	0.003	1	9.820
**2**	111	0.932	0.022	1	8.540
**3**	85	1.035	0.017	1	6.580
**4**	161	1.032	0.032	1, 4	4.446
**5**	574	1.095	0.115	1-4, 6-8, 10	2.854
**6**	1194	0.836	0.239	1-10	1.251
**7**	34	0.916	0.007	2	9.650
**8**	43	0.827	0.009	2	8.880
**9**	82	0.961	0.016	2	7.630
**10**	39	0.771	0.008	2	6.420
**11**	19	0.670	0.004	2	5.840
**12**	18	0.713	0.004	2	5.470
**13**	81	0.839	0.016	2	4.478
**14**	13	0.716	0.003	3	9.860
**15**	23	0.975	0.005	3	9.500
**16**	40	1.058	0.008	3	8.870
**17**	93	1.032	0.019	3	7.540
**18**	105	0.977	0.021	3	5.560
**19**	57	0.752	0.011	3	3.940
**20**	96	1.063	0.019	4	9.030
**21**	48	0.851	0.010	4	7.590
**22**	122	0.967	0.024	4	5.890
**23**	56	1.037	0.011	5	9.430
**24**	110	1.047	0.022	5	7.770
**25**	67	0.720	0.013	5	6.000
**26**	89	0.840	0.018	5	4.440
**27**	25	0.742	0.005	6	9.740
**28**	40	0.863	0.008	6	9.090
**29**	33	0.888	0.007	6	8.360
**30**	99	1.031	0.020	6	7.040
**31**	135	1.083	0.027	6	4.700
**32**	65	0.999	0.013	7	9.340

**33**	18	0.833	0.004	7	8.510
**34**	16	0.692	0.003	7	8.170
**35**	70	0.955	0.014	7	7.310
**36**	169	1.088	0.034	7, 8	5.621
**37**	140	1.047	0.028	7, 8	4.061
**38**	43	1.076	0.009	8	9.560
**39**	34	0.794	0.007	8	8.790
**40**	36	0.844	0.007	8	8.090
**41**	71	0.927	0.014	8	7.020
**42**	62	1.046	0.012	9	9.370
**43**	41	0.704	0.008	9	8.340
**44**	100	0.931	0.020	9	6.930
**45**	92	0.820	0.018	9	5.010
**46**	44	0.624	0.009	9	3.650
**47**	77	1.049	0.015	10	9.220
**48**	24	0.712	0.005	10	8.210
**49**	68	0.916	0.014	10	7.290
**50**	121	0.981	0.024	10	5.400

***Calculated as the average RMSD of all pairs of arrangements in the cluster.**

Table S3: Number of structures (members) in each of the 50 generated clusters and average over the pairwise RMSD between these members. Additionally, the simulations, from which snapshots are included in a specific cluster, and the target RMSD averaged over all cluster members are given.
